# Self‐Healing Mechanism of Lithium in Lithium Metal

**DOI:** 10.1002/advs.202105574

**Published:** 2022-02-25

**Authors:** Junyu Jiao, Genming Lai, Liang Zhao, Jiaze Lu, Qidong Li, Xianqi Xu, Yao Jiang, Yan‐Bing He, Chuying Ouyang, Feng Pan, Hong Li, Jiaxin Zheng

**Affiliations:** ^1^ School of Advanced Materials Peking University Shenzhen Graduate School Shenzhen 518055 P. R. China; ^2^ Shenzhen All‐Solid‐State Lithium Battery Electrolyte Engineering Research Center Institute of Materials Research (IMR) Tsinghua Shenzhen International Graduate School Shenzhen 518055 P. R. China; ^3^ School of Materials Science and Engineering Tsinghua University Beijing 100084 P. R. China; ^4^ Beijing Key Laboratory for New Energy Materials and Devices Institute of Physics Chinese Academy of Sciences Beijing 100190 P. R. China; ^5^ Fujian Science & Technology Innovation Laboratory for Energy Devices of China (21C‐LAB) Ningde 352100 P. R. China

**Keywords:** dendrite growth, dendrite morphology, Li deposition, molecular dynamic simulation, neural network, potential, self‐healing

## Abstract

Li is an ideal anode material for use in state‐of‐the‐art secondary batteries. However, Li‐dendrite growth is a safety concern and results in low coulombic efficiency, which significantly restricts the commercial application of Li secondary batteries. Unfortunately, the Li‐deposition (growth) mechanism is poorly understood on the atomic scale. Here, machine learning is used to construct a Li potential model with quantum‐mechanical computational accuracy. Molecular dynamics simulations in this study with this model reveal two self‐healing mechanisms in a large Li‐metal system, viz. surface self‐healing, and bulk self‐healing. It is concluded that self‐healing occurs rapidly in nanoscale; thus, minimizing the voids between the Li grains using several comprehensive methods can effectively facilitate the formation of dendrite‐free Li.

## Graphical abstract

Li is an ideal anode material for use in state‐of‐the‐art secondary batteries. A deep neural network is adopted to construct a Li potential with quantum‐mechanical computational accuracy. Using this potential, this study achieves a large‐scale Li–metal system simulation and reveals two self‐healing mechanisms, viz. Li surface self‐healing and Li bulk self‐healing.

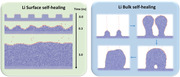

## 1 Introduction

Li metal has a low electrochemical potential (3.04 V vs standard hydrogen electrode) and a specific capacity of ≤3860 mAh g^−1^, making it an ideal anode material for next‐generation secondary batteries.^[^ ^1^, ^2^, ^3^ ^]^ However, the commercial application of Li secondary batteries is hampered by safety concerns and low coulombic efficiency:^[^ ^4^, ^5^ ^]^ the battery can short circuit if the separator is penetrated by uncontrolled Li‐dendrite growth,^[^ ^6^ ^]^ while the coulombic efficiency is reduced by the formation of “dead Li” during cycling.^[^ ^7^ ^]^ Several characterization methods have been used to elucidate the dynamic behavior of Li‐metal deposition.^[^ ^8^, ^9^ ^]^ Although researchers have generally agreed on the atomic‐scale behavior of Li‐dendrite growth, most experimental studies have only described this phenomenon, without providing a comprehensive understanding.^[^ ^10^, ^11^ ^]^ For instance, the critical current density (CCD) is commonly used to determine whether dendritic growth will occur;^[^ ^12^, ^13^ ^]^ in other words, it is considered that Li dendrites will only grow above the CCD. However, there is evidence that Li can repair itself and inhibit dendrite formation when the current density is sufficiently high.^[^ ^14^ ^]^ Additionally, it is widely accepted that the dendrite diameter increases with increasing temperature; however, self‐healing sometimes occurs when the current density is greater than ≈9 mA cm^−2^ or when the temperature is ≈60 °C.^[^ ^14^, ^15^ ^]^ These phenomena are difficult to explain and require further study.

The Li‐metal deposition mechanism has been studied using several numerical simulation methods, including finite element simulations,^[^ ^16^, ^17^, ^18^, ^19^, ^20^ ^]^ ab initio calculations,^[^ ^21^, ^22^, ^23^ ^],^ and molecular dynamics simulations.^[^ ^14^, ^24^, ^25^ ^]^ Although finite element simulation can be performed at a large scale and with a long simulation time, it cannot reflect the movement of Li atoms during deposition owing to its reliance on partial differential equations.^[^ ^16^ ^]^ Additionally, while ab initio calculations accurately reflect the atomic‐scale kinetic and thermodynamic properties of Li,^[^ ^22^ ^]^ their high computational cost limits the simulation scale to hundreds of atoms and several picoseconds.^[^ ^26^ ^]^ Consequently, they are not suitable for analyzing the emergence phenomenon of Li deposition, such as dendrite formation. Molecular dynamics simulations overcome the limitations of ab initio calculations due to the larger scale and higher speed; however, discrepancies remain between predictions based on classical Li potentials and experimental results.^[^ ^27^, ^28^ ^]^ Meanwhile, machine learning is an emerging and powerful technology that can be used to optimize potential function parameters with an accuracy approaching that of quantum‐mechanics computations.^[^ ^29^, ^30^, ^31^ ^]^ Recently, machine‐learning‐based potentials were successfully applied to several systems, including N_2_O_5_ in atmospheric aerosol,^[^ ^32^ ^]^ methane combustion,^[^ ^33^ ^]^ and disordered silicon.^[^ ^34^ ^]^

Herein, we studied the Li atoms’ behavior in the Li metal battery and investigated the origin of Li dendrite. We developed a Li potential model based on a deep potential neural network to achieve a large‐scale (>100 000 atoms) simulation. The model predictions are close to those of ab initio calculations and experimental measurements. By molecular dynamics simulation based on our Li model, two types of self‐healing mechanisms are revealed: surface and bulk self‐healing. We further studied the morphology of Li dendrite and identified three different Li‐dendrite shapes: “needle,” “mushroom,” and “hemisphere.” We finally introduce the concept of local current density (LCD) as a supplement to CCD and propose comprehensive straights to accelerate Li self‐healing.

## 2 Surface Self‐Healing

**Figure 1:**
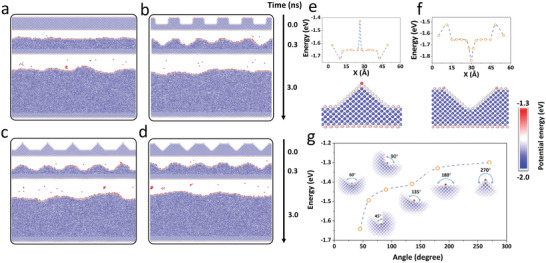
Li homogeneous deposition and surface self‐healing. Snapshots at 0, 0.3, and 3.0 ns when the initial surface had a) a flat configuration, b) rectangular defects, c) triangular defects, and d) inverted triangular defects. Potential energy distribution of Li atoms with e) upright triangular configuration, f) inverted triangular configuration, and g) valley configuration with six different angles (45°–270°). Different colored balls correspond to different potential energies, as indicated by the color scale bar.

We developed a Li surface deep potential model (Li‐SP) using a machine learning algorithm with the same accuracy as ab initio calculations during robust testing (see Experimental Section, Figures S1–S9, and Table S1, Supporting Information, for details of model investigation). The melting point of Li predicted by the Li‐SP is 451.6 K, which is close to the experimental value of 454 K.^[^ ^35^ ^]^ Furthermore, since the surface morphology is critical for Li deposition,^[^ ^17^, ^22^, ^36^ ^]^ we studied the dynamic behavior of Li deposition with different initial atomic‐scale surface morphologies (Figure S10, Supporting Information). Four surface morphologies were constructed for the initial molecular dynamics structures: flat surface, rectangular surface, positive triangular surface, and inverted triangular surface, as shown in the top panels of **Figure** **1**a–d, respectively. Interestingly, all the surface defects were gradually filled during the homogeneous deposition process to form smooth surfaces similar to that of the flat surface (Figure 1b–d; Video S1, Supporting Information). Notably, despite the different surface defects, each surface first evolved to have a corrugated shape after ≈0.3 ns, which is indicative of a more stable surface state. To verify this, we relaxed the rectangular configuration at 300 K without depositing Li atoms and noted that the relaxed surface structure also had a corrugated morphology (Figure S11, Supporting Information). Further research on the effects of temperature, generation rate (*R*_g_), and supercell shape on homogeneous deposition revealed that they all negligibly influenced the final morphology (Figures S12–S15, Supporting Information).

The above‐mentioned results suggest that Li metal possesses inherent surface self‐healing properties during homogeneous deposition. Hence, to clarify the surface self‐healing mechanism, we calculated the atomic potential energies of the different constructions. We noted that the potential energy of the Li atoms in the bulk was generally lower than that on the surface, which indicates that the surface atoms are more active (Figure 1e,f). When only the surface atoms are compared, the potential energy is higher for the atoms at the tips (−1.43 and −1.53 eV) and lower in the valleys (−1.72 and −1.84 eV in the upper subgraph of Figure 1e,f, respectively) of the triangular surface. The primary difference between the tips and valleys is the angle formed by the local atoms. Therefore, to explore the effect of the angle on the potential energy, we placed a Li atom in a valley with various angles, which revealed that the potential energy increased with increasing angles (Figure 1g). When a Li atom was placed on a sloped surface near the bottom of the valley, it relaxed to the bottom and remained stable (Video S2, Supporting Information). These results indicate that the surface self‐healing mechanism originates in the varying potential energies of the Li atoms at various surface morphologies. This ability to self‐heal is inherent in Li, which means the Li surface defects could be smoothed automatically even without Li deposition (Figure S11, Supporting Information).

The Li surface healing experiments confirmed this phenomenon. Atomic force microscope (AFM) images clearly revealed the evolution of the Li surface defects (see Experimental Section). Figure S16a,b (Supporting Information) shows the morphology and depth of the surface defects at 0 and 125 min, respectively. It is obvious that nearly all the defects become shallow, and some small defects even disappear (such as #9 in Figure S16a,b, Supporting Information). In Figure S16c,d (Supporting Information), we show the depth change in lines L1 and L2, on which eight defects (#1 to #8) coincidentally distributed. The difference in the lines between 0 and 125 min was that the lines at 125 min were smooth and the *Z*‐values of the local minimal points were increasing. To provide a more reliable measurement of the defect depth, the depth of a defect was defined as the difference of the altitude between the local maximal and minimal positions near the defect (Figure S17, Supporting Information). For each defect, we drew three lines with different directions to measure the depth of the defects. Table S2 (Supporting Information) shows the measurement results and average values. It is clear that all the depth values of the eight defects were smaller at 125 min. The average depth of the eight defects at 0 min was 16.22 nm, which decreased to 6.97 nm at 125 min. The decrease of the depth originates from the different Li potential energy between the tip and valley, leading to a more smooth and shallow surface. These experimental results confirm the existence of Li surface self‐healing, consistent with our simulation results.

## 3 Bulk Self‐Healing

**Figure 2:**
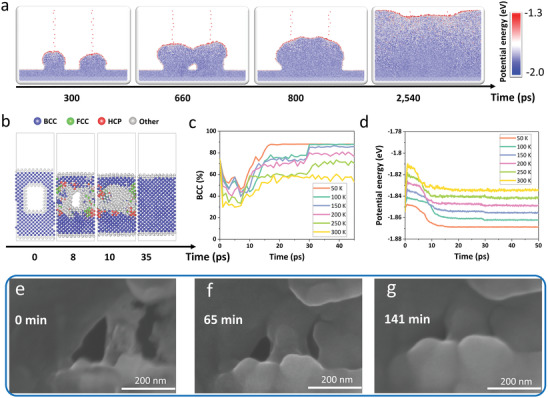
Inhomogeneous deposition and bulk self‐healing. a) Snapshots at 300–2540 ps during inhomogeneous deposition. b) Bulk self‐healing process at 50 K; changes in c) body‐centered cubic (BCC) proportion by adaptive common neighbor analysis and d) potential energy with deposition time at different temperatures during bulk self‐healing. SEM images of defects in the Li foil e) 0 min, f) 65 min, and g) 141 min after the Li foil characterized. The ball colors in (a) denote the potential energy and correspond to the color scale bar. The blue, green, and red balls in (b) denote atoms with local BCC, face‐centered cubic (FCC), and hexagonal close‐packed (HCP) arrangements, respectively, and the grey balls denote other local environments, including surface and amorphous atoms.

Inhomogeneous deposition is electrochemically favored at high current densities, which leads to Li‐dendrite growth.^[^ ^4^ ^]^ This phenomenon is usually considered to be related to the tip effect in the electric field or an uneven electrode surface.^[^ ^17^ ^]^ Hence, we simulated inhomogeneous deposition using the Li‐SP to study the mechanism of dendrite growth and thereby determine a means to suppress it (see Experimental Section and Figure S18, Supporting Information). The results show that two hemispherical dendrites grew separately (300 ps in **Figure** **2**a) until they encountered each other (at ≈660 ps in Figure 2a) to form a larger hemispherical dendrite (at ≈800 ps in Figure 2a). Additionally, a cavity formed between the two dendrites after ≈660 ps, which shrank and then completely disappeared as the two dendrites fuse at ≈800 ps. The large hemisphere fused with other symmetrical hemispheres after further deposition to finally form a smooth surface (at ≈2540 ps in Figure 2a). This phenomenon was different from surface self‐healing and can be referred to as “bulk self‐healing.”

To further study the bulk self‐healing mechanism, we simulated the growth of a Li crystal with a cuboid cavity at 50—300 K (Figure 2b), which revealed that the cavity gradually shrank until it completely disappeared at ≈30 ps, regardless of temperature; adaptive common neighbor analysis (a‐CNA)^[^ ^37^ ^]^ revealed that body‐centered cubic (BCC) states first decreased in number and then increased until self‐healing was complete (Figure 2c). This reveals that some of the Li phase transforms to the amorphous state during deposition and then back to the crystalline state after self‐healing. Amorphous Li, which is more fluid than crystalline Li, as characterized by mean squares displacement (Figure S19, Supporting Information), formed during the self‐healing process at 8–10 ps (Figure 2b), resulting in cavity shrinkage. The change in the potential energy of the Li atoms and the bulk self‐healing process is depicted in detail in Video S3 (Supporting Information). The average potential energy of the Li atoms decreased during the process via an apparently barrierless mechanism, which was the main driving force for cavity shrinkage (Figure 2d); the initial energy increased at ≈1 ps owing to the increase in temperature from 0 K to the target value. Amorphous Li has lower potential energy than that of surface Li and is more fluid than crystalline Li even at a low temperature (Figure S19, Supporting Information). Amorphous Li acts as a lubricative intermediate that triggers bulk self‐healing, resulting in perfect Li. However, it is worth mentioning that this bulk self‐healing phenomenon was not observed in a recent Li‐deposition simulation study.^[^ ^25^ ^]^ The discrepancy is possibly attributable to the different Li potential used (a classical potential for Li), which has a prediction accuracy lower than that of the ab initio calculation.

In order to experimentally validate the simulation results, we conducted Li bulk self‐healing experiments (see Experimental Section). Scanning electron microscope (SEM) images showed that after we created ≈100‐nm‐diameter defects (Figure 2e), the diameter of the defects continuously decreased, and their edges became smoother (Figure 2f). The defects disappeared completely after 141 min (Figure 2g). We recorded the SEM images at room temperature (≈26 °C) and strictly controlled the power and exposure time of the electron beam on the defects of the sample to ensure that the Li foil self‐healed at a temperature far below the melting temperature. This self‐healing was also observed in other Li foil defects (Figure S20, Supporting Information), indicating the universality of this phenomenon. These experiments thus demonstrate that Li metal can indeed experience bulk self‐healing at the nanoscale, thereby validating the predictions of our simulation.

## 4 Dendrite Morphology

**Figure 3:**
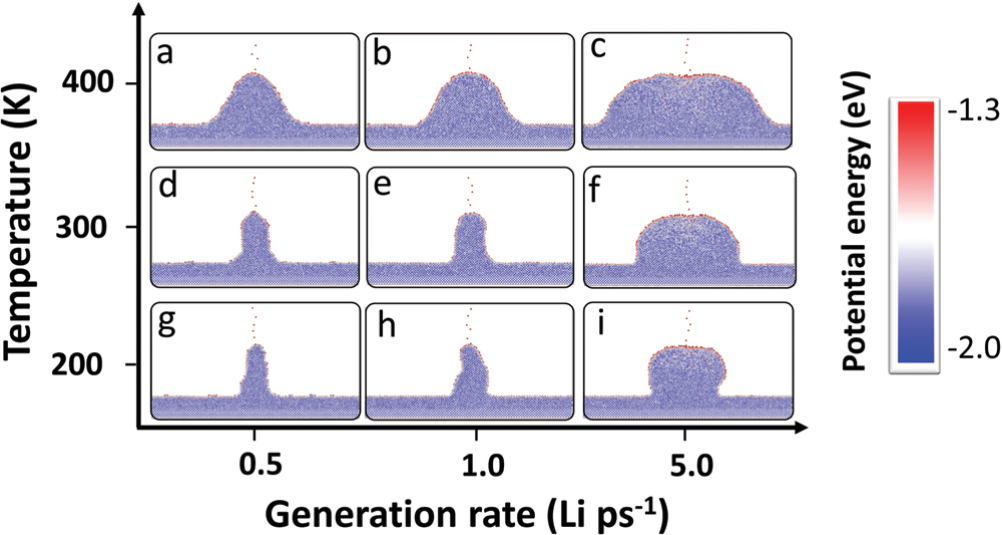
Snapshots of inhomogeneous deposition at different temperatures (*T*) and generation rates (*R*_g_) at a dendrite altitude of 10 nm. *R*_g_ = 0.5 Li ps^−1^ (a, d, g), 1.0 Li ps^−1^ (b, e, h), and 5.0 Li ps^−1^ (c, f, i). a–c) *T* = 400 K, d–f) 300 K, and g–i) 200 K.

Inhomogeneous deposition with the tip effect was simulated at three different temperatures (*T* = 200, 300, and 400 K), and generation rates (*R*_g_ = 0.5, 1, and 5 Li ps^−1^) (see Supporting Information and Experimental Section for simulation conditions). **Figure** **3** shows snapshots of the dendrite at an altitude of 10 nm at various temperatures and generation rates (in contrast, snapshots of the dendrite with ≈6000 deposited Li atoms are shown in Figure S21, Supporting Information). The bottom diameter of the dendrite was seen to increase with temperature at a constant generation rate, while the top diameter increased with increasing generation rate at a constant temperature. At a high generation rate (5 Li ps^−1^; Figure 3g–i), a hemispherical dendrite formed, the bottom of which had noticeable lattice fringes indicative of crystal formation, while the top part remained amorphous. Calculations of the local dendrite temperature show that the temperatures at the bottom and top of the dendrite were significantly different (Figure S22, Supporting Information); the temperature at the bottom was ≈300 K, while that at the surface may have been ≈800 K when the temperature was maintained at 300 K. In contrast, at a low generation rate, the temperature at the top was much closer to that at the bottom. For example, at 0.5 Li ps^−1^, the bottom and top temperatures were ≈300 and 363 K, respectively (Figure S21, Supporting Information). Generally, the high temperature at the dendrite surface is attributable to the energy released by the condensation of the deposited Li atoms, especially at a high generation rate (see Supporting Information for further discussion).

Temperature is an important factor in the deposition process because it controls the fluidities of the bottom and top (surface) atoms, which determine the dendrite morphology. To illustrate this point, the mean squares displacement values of Li on the surface and in the bulk were calculated with temperatures in the range of 50–450 K (Figure S23, Supporting Information), showing that the surface Li atoms were more fluid than those in the bulk, regardless of temperature. All Li atoms were nearly immobile at lower temperatures, whereas the surface atoms were slightly more fluid. Furthermore, the diffusion coefficient of Li on the surface was nearly three orders of magnitude higher than that of the bulk (Figure S24, Supporting Information). Therefore, the above results confirm that the difference in the fluidities of the bottom and top (surface) atoms is critical to the morphology of the dendrite.

Dendrite morphologies can be divided into three types: “needle,” “mushroom,” and “hemisphere.” At a low temperature (e.g., 200 K) and low generation rate, the surface Li atoms are poorly mobile, leading to a “needle” morphology in the deposited area (Figure 3d,g,h). The deposited Li atoms immediately crystallize upon contact with the Li‐metal surface, resulting in a clear lattice fringe (Figure 3g,h). During deposition, the dendrite is frequently fractured because Li atoms constantly collided with the surface, resulting in kinks in the dendrite (Figure S21g and Video S4, Supporting Information), which is consistent with previous observations.^[^ ^38^, ^39^ ^]^ This dendrite fracturing behavior may explain the branched Li‐dendrite morphology observed in these experiments,^[^ ^16^ ^]^ considering that the tips are expected to develop into other dendrites due to the tip effect. The fluidity of the surface Li atoms increases with increasing temperature, leading to a larger area for Li‐atom diffusion; hence, the “needles” have larger diameters at higher temperatures (Figure 3a–c).

At a low temperature and high generation rate, the dendrite favors a “mushroom” morphology owing to the significant difference in the fluidities of the bottom and top atoms (Figure 3i; Figure S20i, Supporting Information). This morphology is attributable to the fact that crystallization occurs easily at the bottom of the dendrite, leading to poor Li fluidity, while the higher surface temperature at the tip results in rapid Li diffusion that forms a “mushroom” morphology.

At a high temperature and high generation rate, Li exists on the surface in a super‐fluid liquid state, leading to a “hemispherical” dendrite (Figure 3c). Contact between two “hemispherical” dendrites triggers the bulk self‐healing mechanism, which suppresses dendrite formation (Figure 2a; Video S5, Supporting Information). Therefore, increasing the fluidity of the surface Li atoms by increasing the temperature and generation rate is an effective method for suppressing dendrite growth.

Our conclusions may be more applicable to solid electrolyte conditions without solid electrolyte interphase (SEI) formation, i.e., a thermodynamically stable interface^[^ ^40^ ^]^; regardless of the fact that dendrites can fuse at high temperature with some special SEIs.^[^ ^15^ ^]^ The mechanism responsible for fusion still warrants further study with respect to the composition, thickness, elasticity, and other SEI factors. While some solid electrolytes have higher shear moduli than Li, studies suggest that they are still unable to inhibit dendrite growth, especially in materials with high ionic conductivities.^[^ ^41^ ^]^ The surface liquid state of Li may significantly contribute to this problem, because a high shear modulus can prevent solid Li‐dendrite formation,^[^ ^2^ ^]^ but cannot prevent liquid Li from flowing in the grain boundaries.

## 5 Discussion

**Figure 4:**
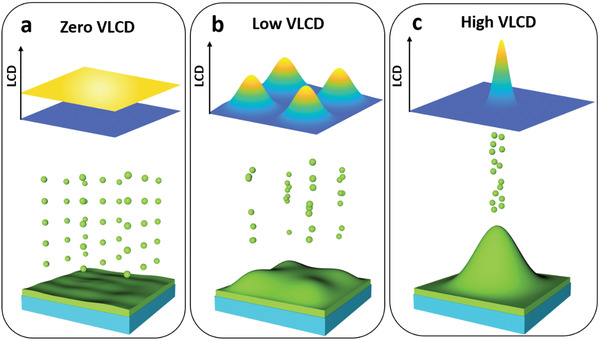
Schematic of Li deposition at different variance of local current density (VLCD). a) Homogeneous deposition with VLCD close to zero, b) inhomogeneous deposition with low VLCD, and c) inhomogeneous deposition with high VLCD.

Although many studies have suggested that the CCD is a crucial factor for dendrite growth,^[^ ^13^, ^41^, ^42^ ^]^ we hypothesize that it is not the decisive factor because it is an average of the current density; for example, a subregion may have a high current density (e.g., tip effect or poor contact) even at a low CCD. Therefore, we further suggest that the LCD distribution and variance in LCD (VLCD) are key factors that affect dendrite growth. For example, Li is mainly deposited homogeneously when the VLCD is close to zero, which triggers surface and bulk self‐healing (**Figure** **4**a). Self‐healing continues to smooth the surface at high CCD and low VLCD (Figure 4b), especially in the solid electrolyte system without the SEI.^[^ ^43^ ^]^ In contrast, inhomogeneous deposition will dominate at a relatively large VLCD, which prevents surface self‐healing (Figure 4c). In this case, the dendrite morphology and the distance between the dendrites are the main factors that affect bulk self‐healing. The larger dendritic “hemispheres” can easily encounter each other, which triggers bulk self‐healing and results in a smooth surface (Figure S25b and Video S5, Supporting Information). We expect that the same process occurs in the case of the “mushroom” dendrites (Figure S25d and Video S6, Supporting Information); however, it is difficult for the “needle” dendrites to encounter each other, which hinders self‐healing (Figure S25c, Supporting Information).

Our simulations are consistent with many previously reported results. An investigation^[^ ^14^ ^]^ of the cycling performance of Li batteries at different current densities revealed that Li develops a smoother surface at a high current density (above ≈9 mA cm^−2^). This high current density results in a high dendrite temperature, which triggers extensive self‐healing both at the surface and in the bulk, thereby smoothing the dendrites and enhancing the cycling performance of the battery. Researchers have also found that Li nuclei increase in size with an increasing deposition temperature or current density,^[^ ^14^, ^15^ ^]^ which is in good agreement with our simulation results. In addition, the time required for Li self‐healing is also related to the size of the defect. In our simulation, the defect size (diameter) in the Li is only ≈1 nm; thus, self‐healing finished in a few nanoseconds. In our experiments, the Li metal defects were ≈100 nm in diameter, and healing finished in hundreds of minutes. In the previous report,^[^ ^14^ ^]^ pores between the dendrites were usually several micrometers, and the fusion of these dendrites took up to 3 days, even at 70 °C. Recently, a long‐cycle‐life all‐solid‐state Li–metal battery was successfully demonstrated through the use of an Ag–C composite.^[^ ^43^ ^]^ The researchers attributed its excellent performance to the uniform Li deposition. We infer that the ultrafast self‐healing of tiny defects formed by the uniform deposition is a key to forming this dendrite‐free deposition morphology.

Thus, instead of suppressing the formation of dendrite, facilitating Li self‐healing is another perspective to improve the performance of Li–metal batteries, which several strategies can achieve. First, the fluidity of the deposited Li atoms can be improved, which is easily achieved by increasing the temperature or introducing amorphous Li, since crystalline (BCC) Li always exhibits an inferior fluidity. Second, a homogeneous deposition environment should be created, thereby lowering the VLCD, reducing the defect size, and quickly completing the Li self‐healing. Third, a solid electrolyte or artificial SEI can be used^[^ ^44^ ^]^ to avoid SEI formation between the Li dendrites, since the SEI as a barrier may prevent the Li self‐healing. Furthermore, a comprehensive plan involving all the above strategies may be more efficient, since they are not mutually exclusive.

## 6 Conclusion

In summary, a machine‐learning‐based Li potential model was developed to achieve the accuracy of quantum mechanics calculations. Then, large‐scaled molecular dynamics simulations revealed the rapid self‐healing process in the Li metal, which was confirmed experimentally. Three Li‐dendrite morphologies were identified under different temperature and *R_g_*, viz. “needle,” “mushroom,” and “hemisphere.” Li deposition simulations also indicated that high fluidity and low VLCD are key to facilitating contact and triggering surface and bulk self‐healing. Thus, charging the Li battery at a relatively high temperature (e.g., increasing *T* to ≈350 K) and modifying the current collector to have a more homogeneous surface, especially in a solid electrolyte system, can potentially suppress dendrite growth. This study introduces a promising strategy for investigating the Li‐deposition mechanism at the atomic level using a machine‐learning derived potential, and provides new perspectives about future research for improving the performance of Li–metal anodes.

## 7 Experimental Section

### DFT Calculations

The Vienna ab initio simulation package (VASP)^[^ ^45^, ^46^ ^]^ based on density functional theory (DFT) was used to generate the training and testing datasets. The generalized gradient approximation (GGA) with a parametrized exchange‐correlation function according to Perdew Burke and Ernzerhof (PBE) was used during calculations.^[^ ^47^ ^]^ The valence electron wave functions were expanded in the plane wave basis sets, and the projector augmented wave (PAW) method was used to describe the core‐electron interactions.^[^ ^48^ ^]^ The plane‐wave cut‐off energy was set to be 380 eV for all calculations after a cut‐off energy test range from 50 to 800 eV. This cut‐off value was sufficient to reproduce accurate results for Li and has a relatively low cost of computing resources. About 10 000 bulk configurations and 6000 surface configurations were selected for the model training (see Supporting Information for DFT calculations and datasets details).

### Training Models

The neural network proposed by Zhang et al.^[^ ^30^, ^49^, ^50^, ^51^ ^]^ was used to develop the Li potential models. In this method, the total energy of the system is the sum of all the single atomic energies: *E* = Σ*E_i_*. The energy of each atom (*E_i_*) was determined by its local environment (other atoms) within a cut‐off radius, defined as *E_i_* = *ϕ*_*ω*_(*D_i_*); where *φ* is the function of the neural network, *ω* is the parameter of the networks, and *D_i_* is the descriptor of the local environment for the *i*th atom. The diversity of configuration space was the key to an effective training model. In this work, an active learning scheme^[^ ^50^, ^51^, ^52^ ^]^ was adopted to generate various configurations and regenerate an effective training data set (see Supporting Information for more detail).

### Molecular Dynamics Simulations

All the molecular dynamics simulations were conducted using LAMMPS.^[^ ^53^ ^]^ All the configurations used in Li deposition were supercells of the primitive BCC cell with an experimental lattice constant of 3.51 Å.^[^ ^27^, ^54^ ^]^ In the deposition simulation, 18 layers of Li atoms were the substrate with Miller indices of (100), and the eight bottom layers of atoms were fixed for a bulk environment. The NVT ensemble with the Langevin thermostat was used in all deposition simulations. A time step of 1 fs was used in all the simulations. All visualizations of the molecular dynamics trajectory were performed with the OVITO program,^[^ ^55^ ^]^ and a‐CNA method^[^ ^37^ ^]^ was used to identify and distinguish a typical phase.

### Homogeneous Deposition Simulation Conditions

The lattice constants of the supercells (Figure S10, Supporting Information) used in this simulation were 28.07 nm × 1.40 nm × 30.00 nm. Li atoms were generated at the top of the supercell, and the probability of atom generation at X and Y coordinates obeyed the distribution of X∼U (0, 28.07) and Y∼U (0, 1.40), where U stands for uniform distribution. The time interval of atom generation (generation rate) was 5 Li ps^−1^, and the falling speed of Li atoms was 500 m s^−1^. These simulations were performed at 300 K by NVT ensemble with a total deposition time of 3 ns.

### Inhomogeneous Deposition Simulation Conditions

The supercells in inhomogeneous deposition had the same dimensions as those in homogeneous deposition (Figure S18, Supporting Information; Figures 2a and 3). Li atoms were generated at the top of the supercell. The generation region was limited to two small regions in Figure S18 (Supporting Information) and Figure 2a, and one small region in Figure 3. The generation rate and the falling rate were the same as the homogeneous deposition conditions as shown in Figure 1a. The simulation with results depicted in Figure 2a was performed at 100 K in a canonical ensemble (NVT) with a total deposition time of 3 ns. In the simulation with results shown in Figure 3, the falling speed of Li was proportional to the generation rate. For example, when the generation rate was 0.5 Li ps^−1^, the falling speed was 500 m s^−1^, and when the generation rate was 1 Li ps^−1^, the falling speed was 1000 m s^−1^, to maintain a constant distance between the two falling atoms.

### Li Surface Healing Experiments

Li–metal foil was prepared with surface defects but without a passivation layer in a glove box with <0.1 ppm of water and oxygen. The depth and morphology of the Li surface defects were then characterized by AFM for several hours. The healing process was performed at room temperature, and the Li foil did not melt during the AFM measurements.

### Li Bulk Healing Experiments

Li metal foil was prepared with a smooth, fresh surface with no passivation layer in a glove box with water and oxygen contents both less than 0.1 ppm. In the glove box, the Li foil was punctured with a tiny needle to create defects with a diameter of ≈100 nm and then transferred to the SEM sample box without being exposed to air. Next, these defects were searched and characterized using SEM. The exposure time for each image was 5—8 s. After recording each image, the electron beam was quickly moved away from the characterized areas to reduce the influence of electron beam irradiation on the Li surface. The same Li defects were then returned to at regular intervals to capture images of these defects, thereby observing the self‐healing process in real time. This process was carried out at room temperature (≈26 °C), and the Li foil did not melt during SEM imaging.

## Conflict of Interest

The authors declare no conflict of interest.

## Author Contributions

J. J. and G. L. contributed equally to this work. J.J., J.L., and J.Z. were associated with conceptualization; J.J. and G.L. were associated with methodology, investigation, and data curation; L.Z., J.L., and Q.L. conducted the experiments; G.L., X.X., and J.J. were associated with study visualization; J.J., G.L., and Y.J. were associated with software; J.J., G.L., and L.Z. were associated with formal analysis; J.Z. and C.O. were associated with funding acquisition; J.Z. was associated with project administration; J.Z. and H.L. supervised the study; J.J. wrote the original draft; J.J., G.L., Y.H., F.P., C.O., H.L., and J.Z reviewed and edited the final manuscript.

## Supporting information

Supporting Information

Supplemental Video 1

Supplemental Video 2

Supplemental Video 3

Supplemental Video 4

Supplemental Video 5

Supplemental Video 6

## Data Availability

The datasets in this study are available at GitHub: https://github.com/msdlabpku/Li-datasets.

## References

[1] J. M. Tarascon , M. Armand , Nature 2001, 414, 359. 11713543 10.1038/35104644

[2] W. Xu , J. Wang , F. Ding , X. Chen , E. Nasybulin , Y. Zhang , J.‐G. Zhang , Energy Environ. Sci. 2014, 7, 513.

[3] X. Shen , X.‐Q. Zhang , F. Ding , J.‐Q. Huang , R. Xu , X. Chen , C. Yan , F.‐Y. Su , C.‐M. Chen , X. Liu , Q. Zhang , Energy Mater. Adv. 2021, 2021, 1205324.

[4] B. Liu , J.‐G. Zhang , W. Xu , Joule 2018, 2, 833.

[5] X.‐B. Cheng , H. Liu , H. Yuan , H.‐J. Peng , C. Tang , J.‐Q. Huang , Q. Zhang , SusMat 2021, 1, 38.

[6] X.‐B. Cheng , R. Zhang , C.‐Z. Zhao , Q. Zhang , Chem. Rev. 2017, 117, 10403. 28753298 10.1021/acs.chemrev.7b00115

[7] P. P. Paul , E. J. McShane , A. M. Colclasure , N. Balsara , D. E. Brown , C. T. Cao , B. R. Chen , P. R. Chinnam , Y. Cui , E. J. Dufek , D. P. Finegan , S. Gillard , W. X. Huang , Z. M. Konz , R. Kostecki , F. Liu , S. Lubner , R. Prasher , M. B. Preefer , J. Qian , M. T. F. Rodrigues , M. Schnabel , S. B. Son , V. Srinivasan , H. G. Steinruck , T. R. Tanim , M. F. Toney , W. Tong , F. Usseglio‐Viretta , J. Y. Wan , et al., Adv. Energy Mater. 2021, 11, 2100372.

[8] Y. He , X. Ren , Y. Xu , M. H. Engelhard , X. Li , J. Xiao , J. Liu , J.‐G. Zhang , W. Xu , C. Wang , Nat. Nanotechnol. 2019, 14, 1042. 31611656 10.1038/s41565-019-0558-z

[9] S. Wang , H. Xu , W. Li , A. Dolocan , A. Manthiram , J. Am. Chem. Soc. 2018, 140, 250. 29250960 10.1021/jacs.7b09531

[10] Y. Sun , T. Yang , H. Ji , J. Zhou , Z. Wang , T. Qian , C. Yan , Adv. Energy Mater. 2020, 10, 2002373.

[11] P. Albertus , V. Anandan , C. Ban , N. Balsara , I. Belharouak , J. Buettner‐Garrett , Z. Chen , C. Daniel , M. Doeff , N. J. Dudney , B. Dunn , S. J. Harris , S. Herle , E. Herbert , S. Kalnaus , J. A. Libera , D. Lu , S. Martin , B. D. McCloskey , M. T. McDowell , Y. S. Meng , J. Nanda , J. Sakamoto , E. C. Self , S. Tepavcevic , E. Wachsman , C. Wang , A. S. Westover , J. Xiao , T. Yersak , ACS Energy Lett. 2021, 6, 1399.

[12] F. Han , A. S. Westover , J. Yue , X. Fan , F. Wang , M. Chi , D. N. Leonard , N. J. Dudney , H. Wang , C. Wang , Nat. Energy 2019, 4, 187.

[13] Y. Lu , C.‐Z. Zhao , H. Yuan , X.‐B. Cheng , J.‐Q. Huang , Q. Zhang , Adv. Funct. Mater. 2021, 31, 2009925.

[14] L. Li , S. Basu , Y. Wang , Z. Chen , P. Hundekar , B. Wang , J. Shi , Y. Shi , S. Narayanan , N. Koratkar , Science 2018, 359, 1513. 29599241 10.1126/science.aap8787

[15] J. Wang , W. Huang , A. Pei , Y. Li , F. Shi , X. Yu , Y. Cui , Nat. Energy 2019, 4, 664.

[16] A. Jana , S. I. Woo , K. S. N. Vikrant , R. E. García , Energy Environ. Sci. 2019, 12, 3595.

[17] S.‐H. Wang , Y.‐X. Yin , T.‐T. Zuo , W. Dong , J.‐Y. Li , J.‐L. Shi , C.‐H. Zhang , N.‐W. Li , C.‐J. Li , Y.‐G. Guo , Adv. Mater. 2017, 29, 1703729. 10.1002/adma.201703729 28891207

[18] J. Wu , Z. Rao , X. Liu , Y. Shen , C. Fang , L. Yuan , Z. Li , W. Zhang , X. Xie , Y. Huang , Adv. Mater. 2021, 33, 2007428. 10.1002/adma.202007428 33543568

[19] X. Xu , Y. Liu , J.‐Y. Hwang , O. O. Kapitanova , Z. Song , Y.‐K. Sun , A. Matic , S. Xiong , Adv. Energy Mater. 2020, 10, 2002390.

[20] Y. Liu , X. Xu , M. Sadd , O. O. Kapitanova , V. A. Krivchenko , J. Ban , J. Wang , X. Jiao , Z. Song , J. Song , S. Xiong , A. Matic , Adv. Sci. 2021, 8, 2003301. 10.1002/advs.202003301 PMC7927631 33717853

[21] M. Jäckle , A. Groß , J. Chem. Phys. 2014, 141, 174710. 25381540 10.1063/1.4901055

[22] M. Jäckle , K. Helmbrecht , M. Smits , D. Stottmeister , A. Groß , Energy Environ. Sci. 2018, 11, 3400.

[23] J. Jiao , R. Xiao , M. Han , Z. Wang , L. Chen , Appl. Surf. Sci. 2020, 515, 145886.

[24] M. Yang , Y. Liu , A. M. Nolan , Y. Mo , Adv. Mater. 2021, 33, 2008081. 10.1002/adma.202008081 33576149

[25] X. Wang , G. Pawar , Y. Li , X. Ren , M. Zhang , B. Lu , A. Banerjee , P. Liu , E. J. Dufek , J.‐G. Zhang , J. Xiao , J. Liu , Y. S. Meng , B. Liaw , Nat. Mater. 2020, 19, 1339. 32719511 10.1038/s41563-020-0729-1

[26] J. Behler , Angew. Chem., Int. Ed. 2017, 56, 12828. 10.1002/anie.201703114 28520235

[27] A. Nichol , G. J. Ackland , Phys. Rev. B 2016, 93, 184101.

[28] J. R. Vella , F. H. Stillinger , A. Z. Panagiotopoulos , P. G. Debenedetti , J. Phys. Chem. B 2015, 119, 8960. 25192474 10.1021/jp5077752

[29] J. Behler , M. Parrinello , Phys. Rev. Lett. 2007, 98, 146401. 17501293 10.1103/PhysRevLett.98.146401

[30] L. Zhang , J. Han , H. Wang , R. Car , W. E. , Phys. Rev. Lett. 2018, 120, 143001. 29694129 10.1103/PhysRevLett.120.143001

[31] A. P. Bartók , M. C. Payne , R. Kondor , G. Csányi , Phys. Rev. Lett. 2010, 104, 136403. 20481899 10.1103/PhysRevLett.104.136403

[32] M. Galib , D. T. Limmer , Science 2021, 371, 921. 33632842 10.1126/science.abd7716

[33] J. Zeng , L. Cao , M. Xu , T. Zhu , J. Z. H. Zhang , Nat. Commun. 2020, 11, 5713. 33177517 10.1038/s41467-020-19497-z PMC7658983

[34] V. L. Deringer , N. Bernstein , G. Csányi , C. Ben Mahmoud , M. Ceriotti , M. Wilson , D. A. Drabold , S. R. Elliott , Nature 2021, 589, 59. 33408379 10.1038/s41586-020-03072-z

[35] in Smithells Metals Reference Book, Vol. 7 (Eds.: E. A. Brandes , G. Brook ), Butterworth‐Heinemann, Oxford 1992.

[36] F. Ding , W. Xu , G. L. Graff , J. Zhang , M. L. Sushko , X. Chen , Y. Shao , M. H. Engelhard , Z. Nie , J. Xiao , X. Liu , P. V. Sushko , J. Liu , J.‐G. Zhang , J. Am. Chem. Soc. 2013, 135, 4450. 23448508 10.1021/ja312241y

[37] A. Stukowski , Modell. Simul. Mater. Sci. Eng. 2012, 20, 045021.

[38] H. Ghassemi , M. Au , N. Chen , P. A. Heiden , R. S. Yassar , Appl. Phys. Lett. 2011, 99, 123113.

[39] A. Kushima , K. P. So , C. Su , P. Bai , N. Kuriyama , T. Maebashi , Y. Fujiwara , M. Z. Bazant , J. Li , Nano Energy 2017, 32, 271.

[40] S. Wenzel , T. Leichtweiss , D. Krüger , J. Sann , J. Janek , Solid State Ionics 2015, 278, 98.

[41] Z. Lu , Z. Yang , C. Li , K. Wang , J. Han , P. Tong , G. Li , B. S. Vishnugopi , P. P. Mukherjee , C. Yang , W. Li , Adv. Energy Mater. 2020, 11, 2003811.

[42] D. Lin , Y. Liu , Y. Cui , Nat. Nanotechnol. 2017, 12, 194. 28265117 10.1038/nnano.2017.16

[43] Y.‐G. Lee , S. Fujiki , C. Jung , N. Suzuki , N. Yashiro , R. Omoda , D.‐S. Ko , T. Shiratsuchi , T. Sugimoto , S. Ryu , J. H. Ku , T. Watanabe , Y. Park , Y. Aihara , D. Im , I. T. Han , Nat. Energy 2020, 5, 299.

[44] Y. Liu , D. Lin , P. Y. Yuen , K. Liu , J. Xie , R. H. Dauskardt , Y. Cui , Adv. Mater. 2017, 29, 1605531. 10.1002/adma.201605531 28032934

[45] G. Kresse , J. Furthmüller , Phys. Rev. B 1996, 54, 11169. 10.1103/physrevb.54.11169 9984901

[46] G. Kresse , J. Furthmüller , Comput. Mater. Sci. 1996, 6, 15.

[47] J. P. Perdew , K. Burke , M. Ernzerhof , Phys. Rev. Lett. 1996, 77, 3865. 10062328 10.1103/PhysRevLett.77.3865

[48] P. E. Blöchl , Phys. Rev. B 1994, 50, 17953. 10.1103/physrevb.50.17953 9976227

[49] H. Wang , L. Zhang , J. Han , W. E. , Comput. Phys. Commun. 2018, 228, 178.

[50] L. Zhang , D.‐Y. Lin , H. Wang , R. Car , W. E. , Phys. Rev. Mater. 2019, 3, 023804.

[51] Y. Zhang , H. Wang , W. Chen , J. Zeng , L. Zhang , H. Wang , W. E. , Comput. Phys. Commun. 2020, 253, 107206.

[52] J. Wu , Y. Zhang , L. Zhang , S. Liu , Phys. Rev. B 2021, 103, 024108.

[53] S. Plimpton , J. Comput. Phys. 1995, 117, 1.

[54] W.‐S. Ko , J. B. Jeon , Comput. Mater. Sci. 2017, 129, 202.

[55] A. Stukowski , Modell. Simul. Mater. Sci. Eng. 2010, 18, 015012.

