# Supplementary material for: Self‐Healing Mechanism of Lithium in Lithium Metal
Source: Adv Sci (Weinh). 2022 Feb 25;9(12):2105574. doi: 10.1002/advs.202105574 (PMC9036043; doi:10.1002/advs.202105574)
Supplement: Supplementary file 1 — Supporting Information [file ADVS-9-2105574-s006.pdf]

Supporting Information for

Self-healing mechanism of lithium in lithium metal

Junyu Jiao†, Genming Lai†, Liang Zhao, Jiaze Lu, Qidong Li, Xianqi Xu, Yao Jiang, Yan-Bing He, Chuying Ouyang, Feng Pan, Hong Li, and Jiaxin Zheng**

†These authors contributed equally to this work.
*Correspondence to: zhengjx@pkusz.edu.cn; hli@iphy.ac.cn;

Contents:

1. Supplementary Methods
2. Supplementary Figures (Figures S1 to S25)
3. Supplementary Table S1 and S2
4. Supplementary References

Other Supplementary Materials for this manuscript includes the following:
Supplementary Videos S1 to S6

Supplementary Methods

S1. Model Generation and Test

DFT calculations and datasets. We used the Vienna ab initio simulation package (VASP) [1] based on density functional theory (DFT) to generate the training and testing datasets. The generalized gradient approximation (GGA) with a parametrized exchange-correlation function according to Perdew Burke and Ernzerhof (PBE) was used during the calculations [2]. The valence electron wave functions were expanded in the plane wave basis sets, and the projector augmented wave (PAW) method was used to describe the core-electron interactions [3]. The plane-wave cutoff energy was set to be 380 eV for all calculations after a cutoff energy test range from 50 eV to 800 eV. This cutoff value is sufficient to reproduce the accurate results for lithium and has a relatively low cost of computing resources.

To have a reliable prediction of the lithium surface properties, we generated two data sets: bulk data and surface data. The basic structure we used in the bulk data is a supercell of 4×4×4 larger than the primitive bcc cell. The primary surface structures contained three low Miller index surfaces, which are (1 0 0), (1 1 0), and (1 1 1), respectively. The surface structures were terminated by more than a 20 Å vacuum interval. The Brillouin zone samplings were performed using a 3×3×3 k-point grid in the Monkhorst-Pack scheme for the bulk structure and 3×3×1 for the surface structures. The convergence precision of electron self-consistent field calculation was less than 10^{-7} eV. Totally, about 10,000 bulk configurations and 6,000 surface configurations were selected for the model training.

The ab initio molecular dynamics (AIMD) generated the testing data sets in a canonical ensemble (NVT) with temperatures ranging from 200 K to 1,000K. Three supercells of 4×4×4 larger than the primitive bcc cell with randomly disturbed were used as the initial configurations. The total time steps of AIMD were 15,000, and 1,500 configurations were selected for the bulk test dataset. The initial surface configurations contained three low Miller index surfaces, same with training datasets, with total AIMD time steps of 12,000 and 1,000 configurations were selected for the surface test dataset.

Training models. We used the neural network to develop the Li potential models proposed by Zhang et al [4-6]. In this method, the total energy of the system is the sum of all the single atomic energy: $E = \Sigma E_i$. The energy of each atom (E_i) is determined by its local environment (other atoms) within a cutoff radius, defined as $E_i = \varphi_\omega(D_i)$, where φ is the function of the neural network, ω is the parameter of the networks, and D_i is the descriptor of the local environment for the ith atom. This local environment is usually complex, nonlinear, and has the characteristics of many-body interaction. However, the high-dimensional neural network has a robust fitting ability and can establish the map between the local atomic information and atomic energy (forces) through appropriate training.

The smooth version of the deep neural network model (DP-SE) was employed in our work through the DEEPMD-Kit Python package [5, 7]. The model includes two networks: the embedding network and the fitting network. Before the embedding network, a local coordinate is first established for each atom, and the position information of the local environment within the cutoff radius can be obtained. This position information includes the distance of the surrounding atoms to the target atom (reciprocal) in the three dimensions (reciprocal). This position information defined as D_i are then inputted to the embedded network as the descriptor of the target atom for further processing to ensure the rotation, translation, displacement invariance, and continuity of DP-SE. The cutoff radius was set to 6.8 Å. Both the embedding network and the fitting network use the ResNet-like architecture [8]. The loss function of DP-SE was defined as:

$$L = P_e \Delta E^2 + \frac{P_f}{3N} \sum_i |\Delta F_i|^2 + \frac{P_\xi}{9} \left| |\Delta \xi| \right|^2 \quad (1)$$

where Δ denotes the difference between the DP-SE prediction and the training data, *N* is the number of atoms, *E* is the energy per atom, F_i is the atomic force of atom i, and ξ is the virial tensor divided by *N*. P_e, P_f, and P_ξ are tunable prefactors. Here we increase both P_e and P_ξ from 0.02 to 1, and decreases P_f from 1,000 to 2. The network parameters are updated by the backpropagation algorithm in training (the Adam optimization strategy is adopted [9]). The energy and force results with the decrease of L will be close to those calculated by the DFT.

The Active Learning. The diversity of configuration space is the key to an effective training model. In our work, we adopted an active learning scheme[6, 10] to generate various configurations and regenerate an effective training data set. Active learning contains three steps, which are exploration, labeling, and training. These steps will repeat automatically until the accurate models are established (Figure S1). In the exploration step, we use four different deep neural network potential (DP) models (with varying initialization parameters) to calculate the energy and force for a configuration and compare the difference from these models. We define the model deviation *ε* as the maximal standard deviation of the atomic force predicted by the model ensemble:

$$\varepsilon = \max_i \sqrt{\langle \|f_i - \langle f_i \rangle\|^2 \rangle} \quad (2)$$

Where *i* runs through the atomic indices in a configuration, and the ensemble average ⟨..⟩ is taken over the ensemble of models. When a small *ε* value is reached, all the models give a uniform result for each interatomic force in the configuration. This indicates that the configuration is near the existing training set and can be accurately predicted by all the models. Thus, such configurations will not be labeled.

If *ε* is relatively large, it means that the model ensemble has not been effectively trained near the configuration (possibly due to the lack of diversity in the training data set). Therefore, it is necessary to label this configuration for DFT calculation and put it in the training data set. However, a much large *ε* may also mean that the configuration itself is unreasonable. Thus, we introduce two thresholds and only label the configuration when 0.02 eV/Å < ε < 0.2 eV/Å. For the labeled configurations, we performed DFT calculations to get accurate energy and force results. The new training dataset was added to the previous training datasets to retrain the models. In the next iteration of the training step, we adopted the transfer learning strategy. That is, the initial parameters are not initialized randomly, but the model parameters of the last training are used. The above three steps will be repeated until every *ε* of all configurations is less than 0.02 eV/ Å.

Model test. We developed a lithium deep surface potential model (Li-SP) by ML algorithm, realizing the accuracy of ab initio calculations. Meanwhile, Li-SP can achieve a large-scale simulation of over 100,000 atoms with linear computational costs as system size (using a GPU of NVIDIA Tesla V100 32G). To measure the accuracy of Li-SP, we first calculated the mean absolute error (MAE) of the energy and force between Li-SP and DFT calculation results, respectively, based on the test datasets (including bulk and surface datasets, see Supplementary S2). The energy MAE is 0.85 meV/atom in the bulk test dataset and 1.21 meV/atom in the surface test dataset (Figure S2). The average MAEs of force in three dimensions are 0.019 eV $Å^{-1}$ in the bulk test dataset and 0.015 eV $Å^{-1}$ in the surface test dataset (Figure S3). The equation of state (EOS) of Li calculated by different methods (Li-SP, EAM[11], MEAM[12], and DFT) shows that the EOS of Li-SP are almost perfectly consistent with the EOS of DFT (Figure S4). By contrast, the EOS of EAM and MEAM show a significant deviation from the DFT results. Besides, the radial distribution function (RDF) of lithium at 700 K also shows that the RDF of Li-SP is consistent with the DFT results, more accurate than the results of MEAM and EAM (Figure S5). The melting point of lithium

predicted by Li-SP in the solid-liquid mixed-phase (NVE ensemble) is 451.6 K (Figure S6), which is very close to the experimental value of 454 K [13]. From this point (table. S1), Li-SP is the most reliable potential among the Li potentials [11, 12, 14]. The mean square displacement (MSD) of Li at 1,000 K was also calculated by DFT and Li-SP, and no obvious difference between the two methods (Figure S7). Table S1 lists the predicted values of Li properties by different methods and the comparison with experimental results. It is remarkable that the error between Li-SP and DFT is the smallest in terms of surface energy. All the above results indicate that the Li-SP has the same accuracy as DFT calculations, and it can provide reliable results during the large-scale simulation of lithium deposition.

To highlight the accuracy of Li-SP in the calculation of surface configuration, we also tested Li bulk potential (Li-BP), which has similar networks with Li-SP but was trained without the surface training set. The results were shown in Figures. S8 and S9 and Table S1. The MAE of surface energy test in Li-BP is 2.39 meV/atom (Figure S8), much higher than 1.21 meV/atom in Li-SP. The MAE of surface force test of Li-BP in the Z direction is 0.033 eV $Å^{-1}$ (Figure S9c), which is much higher than that of Li-SP (0.014 eV $Å^{-1}$). These results indicate that Li-SP has more accurate results involved in surface configurations.

S2. The Molecular Dynamic (MD) Simulations

All of the MD simulations were conducted using LAMMPS [15]. All configurations used in Li deposition were supercells of the primitive bcc cell with an experimental lattice constant of 3.51 Å [11, 14]. In the deposition simulation, eighteen layers of Li atoms are the substrate, with eight bottom layers of atoms fixed for a bulk environment. The NVT ensemble with the Langevin thermostat was used in all deposition simulations. A time step of 1 fs was used in all simulations. All visualizations of the MD trajectory were performed with the OVITO program [16], and the adaptive common neighbor analysis (a-CNA) method [17] was used to identify and distinguish a typical phase.

Homogeneous deposition simulation conditions. The size of the supercells (Figure S10) used in this simulation is 28.07 nm × 1.40 nm × 30.00 nm. Li atoms are generated at the top of the supercell, and the probability of atoms generation at X and Y coordinates obeys the distribution of X~U (0, 28.07) and Y~U (0, 1.40), where U stands for uniform distribution. The time interval of atom generation (generation rate) is 5 Li ps^{-1}, and the falling speed of lithium atoms is 500 m s^{-1}. These simulations were performed in a 300 K of NVT ensemble with a total deposition time of 3 ns.

Inhomogeneous deposition simulation conditions. The size of the supercells in inhomogeneous deposition is the same as the size in the homogeneous deposition (Figure S16, 2a, and 3). Lithium atoms were generated at the top of the supercell. The generation region was limited to two small regions in Figure S16 and 2a and one small region in Figure 3. The generation rate and the falling rate are the same as homogeneous deposition conditions in Figure 2a. The simulation of Figure 2a was performed in a 100 K of NVT ensemble with a total deposition time of 3 ns. In Figure 3, the falling speed of Li is proportional to the generation rate. For example, when the Gr is 0.5 Li ps^{-1}, the falling speed is 500 m s^{-1}. When 1 Li ps^{-1}, the falling speed is 1,000 m s^{-1} to keep the distance between the two falling atoms.

S3. Further investigated on homogeneous deposition.

The temperature of the system was controlled at 100 K, 200 K, and 300 K, respectively, while other deposition conditions remain unchanged. The results show that the temperature has little effect on the final Li morphology in the homogeneous deposition (Figures S12 and

S13), except that higher temperature generally results in a smoother surface (Figures S12c and S13c). We analyzed the local structure of Li atoms through the adaptive common neighbor analysis (a-CNA) method [17] and found that temperature would affect the degree of Li crystallization (Figure S13). At low temperature (~100 K), body-centered cubic (BCC) states occupy up to 92.7% of total states (Figure S10b), while at high temperature (~300 K), the amorphous state increases with the BCC state decreasing to 66.3% (Figure S13d). We also studied the influence of Gr on the morphology during deposition, which can be considered as current densities. The Gr are 1 Li ps^{-1}, 5 Li ps^{-1}, 20 Li ps^{-1} with the deposition duration of 15 ns, 3 ns, 0.75 ns, respectively, to ensure the same number of deposited atoms. The results (Figure S14) show that the Gr has little effect on the morphology in the deposition, only the less BCC state at a high Gr (Figure S14c), related to the increased temperatures of the surface. Besides, the simulation with a larger 3D supercell also shows a surface self-healing process in the deposition, indicating the independence of the supercell shape (Figure S15).

S4. Further discussion on inhomogeneous deposition

Discussion on coulombic interaction. Coulombic interaction in the electric field was not considered in the simulation due to the limitation of the model. To overcome this deficiency, we simulate the dendrite growth process by limiting the deposition area, corresponding to the tip effect. Li ions in the electrolyte are affected by a relatively strong electric field in the actual deposition process. Once Li ions are deposited on the surface of lithium metal, Li ions will become Li atoms, and the influence of the electric field for Li atoms will be much weaker than that for the Li ions. Therefore, only considering the interaction between atoms can still give reliable simulation results [18]. Besides, we are focusing on studying the growth process of Li (dendrite) and the dynamic proprieties of Li atoms but not the falling process of Li ions. Therefore, our model can still give reliable simulation results without considering the effect of the electric field.

Discussion on current density. In the actual deposition process, the current density is much lower than this corresponding value of the generation rate (Gr, 1 Li ps^{-1} at an area of 39.3×10^{-14} cm^2 corresponding to ~4×10^{8} mA cm^{-2}). Due to the limitation of simulation time, we cannot adjust the Gr (current density) to an order of magnitude close to the experimental value. Similar situations also exist in other Li deposition (growth) processes [18, 19]. Fortunately, we can still change the Gr within a certain range to study the influence on the dendrite shapes and deduce the dendrite change trend.

Discussion on the surface temperature. The higher surface temperature than the bulk comes from the condensation of deposited Li. The temperature may be overestimated than that of the actual deposition process, considering the heat exchange between the surface and electrolyte. In the actual deposition process, the energy released by every deposited Li is related to the overpotential. Generally, an overpotential of −0.5 V is able to reach a relatively high current density leading to additional energy of 0.5 eV for every Li [20]. When Li ions are reduced to Li atoms at the surface, these energies that Li gained from the overpotential would release as the joule heat leading to the increase of the surface temperature. Suppose all of the energy is converted to kinetic energy on the surface (without considering that some of energy many consume in the electrolyte), the surface will have a temperature of 3,865 K without considering the exchange of heat, according to the thermodynamic formula: $E_k = 3/2\ kT$, where E_k is the kinetic energy, k is the Boltzmann constant, and T is the temperature. However, heat exchange exists between the surface and the local environment (including the SEI, electrolyte, and lithium metal, et al.). Meanwhile, part of the overpotential energy is consumed in the electrolyte and the counter electrode. In our simulation, the heat exchange

only existed between the dendrite and the substrate. Thus, when considering the heat exchange in the systems, this ultra-temperature less likely exists in the liquid electrolyte but more likely exists in the solid electrolyte systems, which is more similar to our simulation environment. The above discussion indicates that the surface temperature is usually higher than the bulk temperature in deposition and may reach the melting point at a high overpotential.

Supplementary Figures

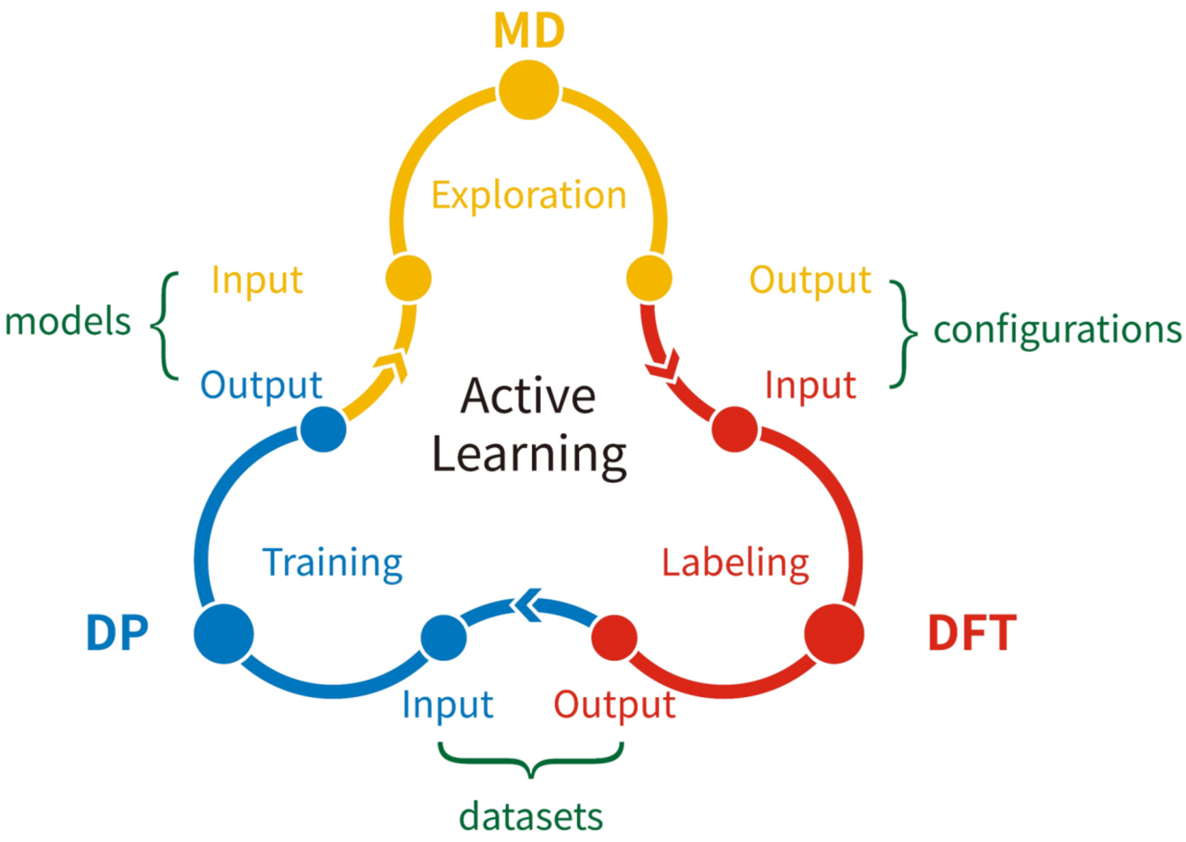

Figure S1. The schematic of active learning.

The active learning contains three steps: exploration, labeling, and training. The DP stands for deep neural network potentials, MD stands for molecular dynamics simulation, and DFT stands for density functional theory calculations. These steps will repeat automatically until the accurate models are established.

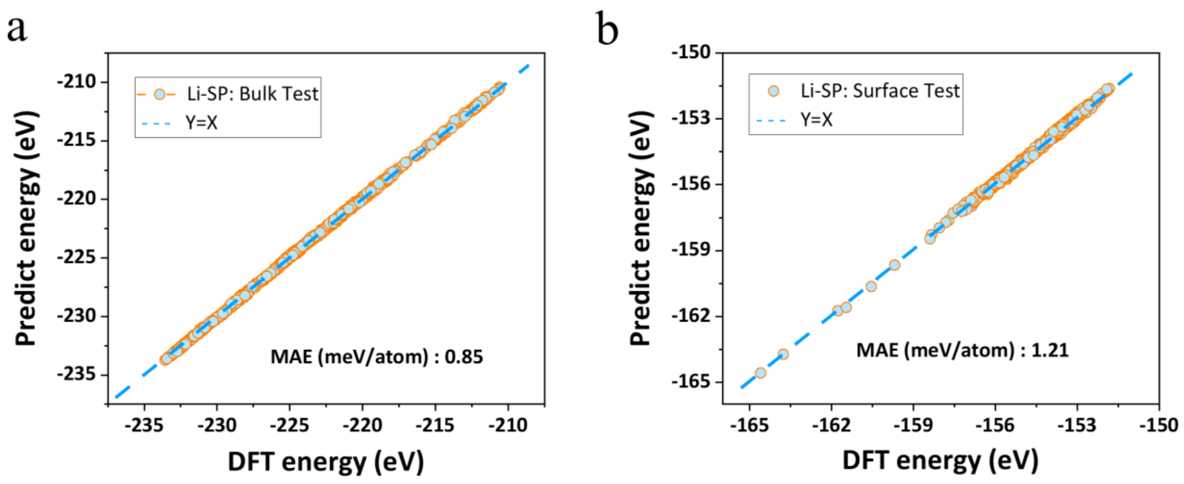

Figure S2. The energy results of the surface and bulk test datasets predicted by Li-SP and DFT calculation.

The MAE is 0.85 meV/atom for bulk test (**a**) and 1.21 meV/atom for surface test (**b**).

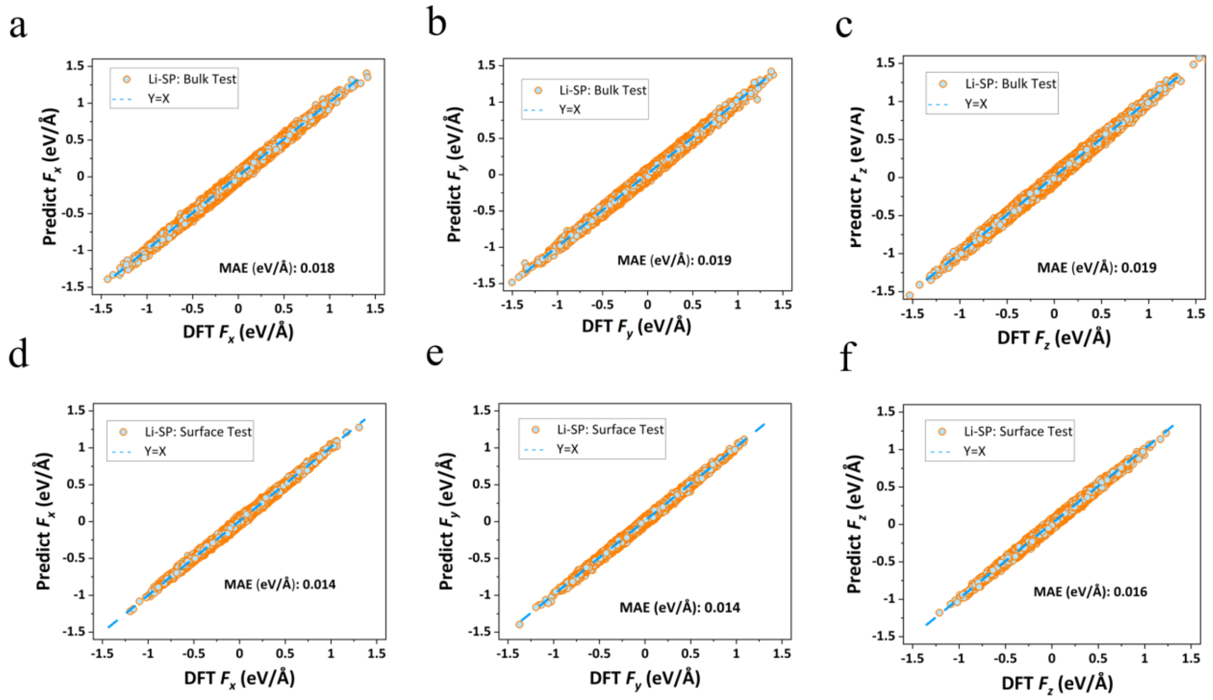

Figure S3. The force results of surface and bulk test dataset predicted by Li-SP and DFT calculation in x, y, and z direction.

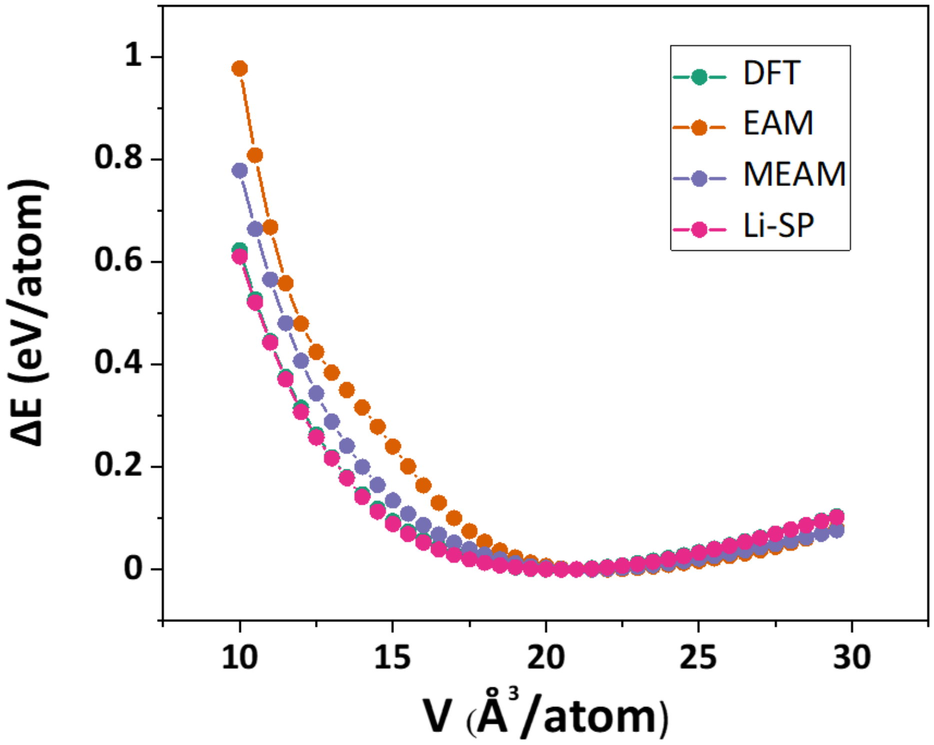

Figure S4. The Li Equation of state (EOS) obtained by different methods.

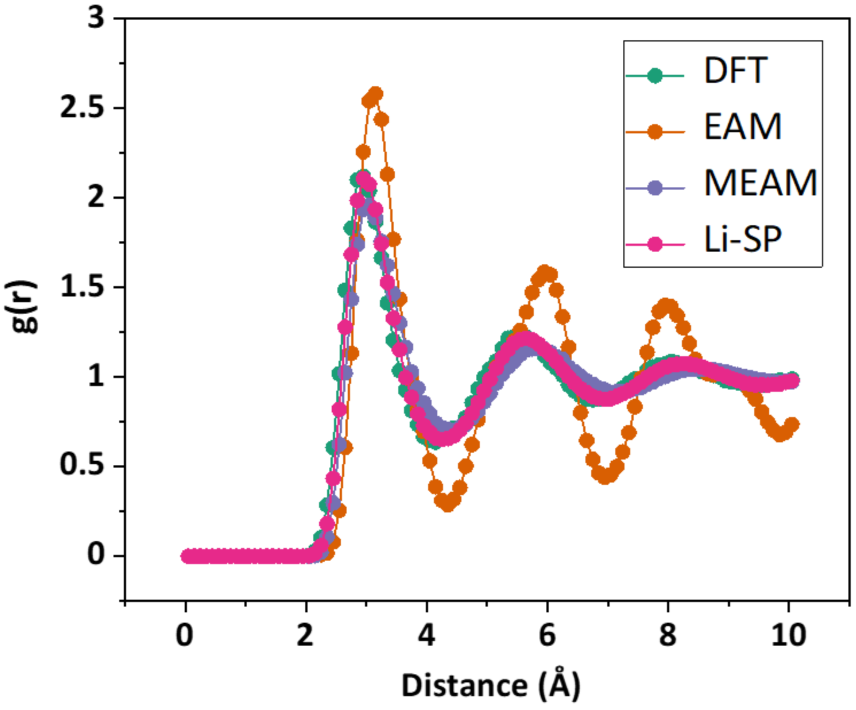

Figure S5. The radial distribution functions of Li measured at 700 K by the above methods.

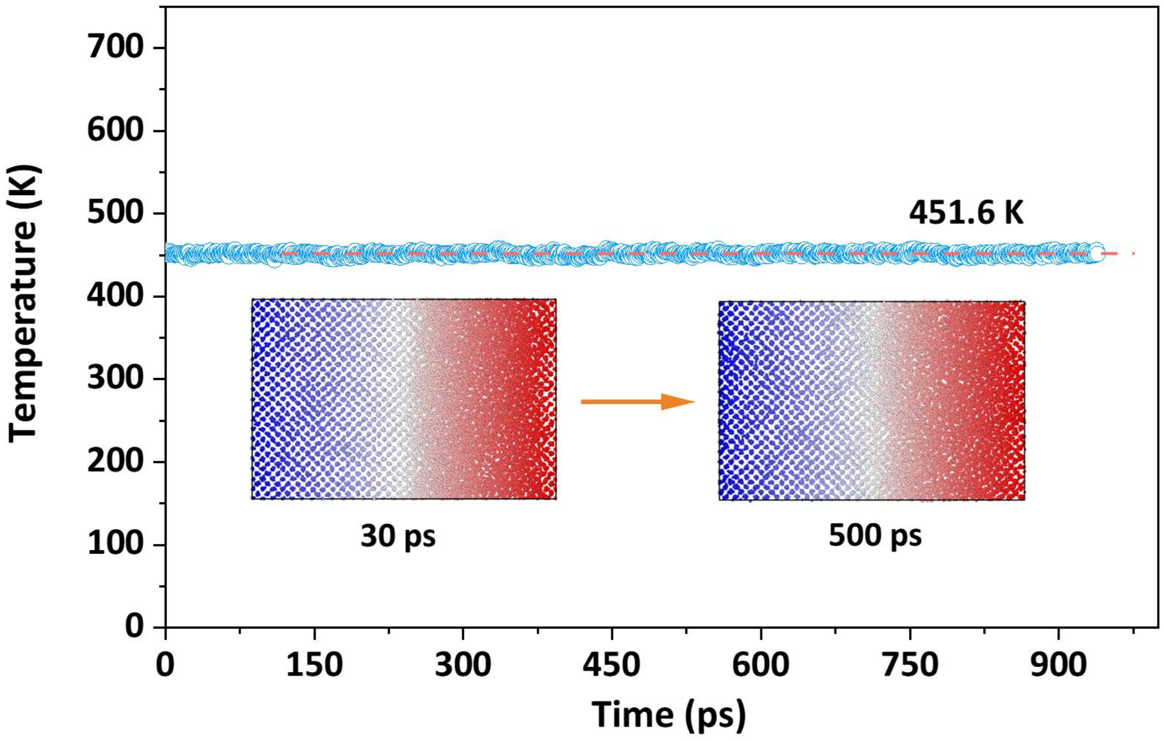

Figure S6. The melting point (451.6 K) of Li measured in solid-liquid mixed phase by Li-SP model.

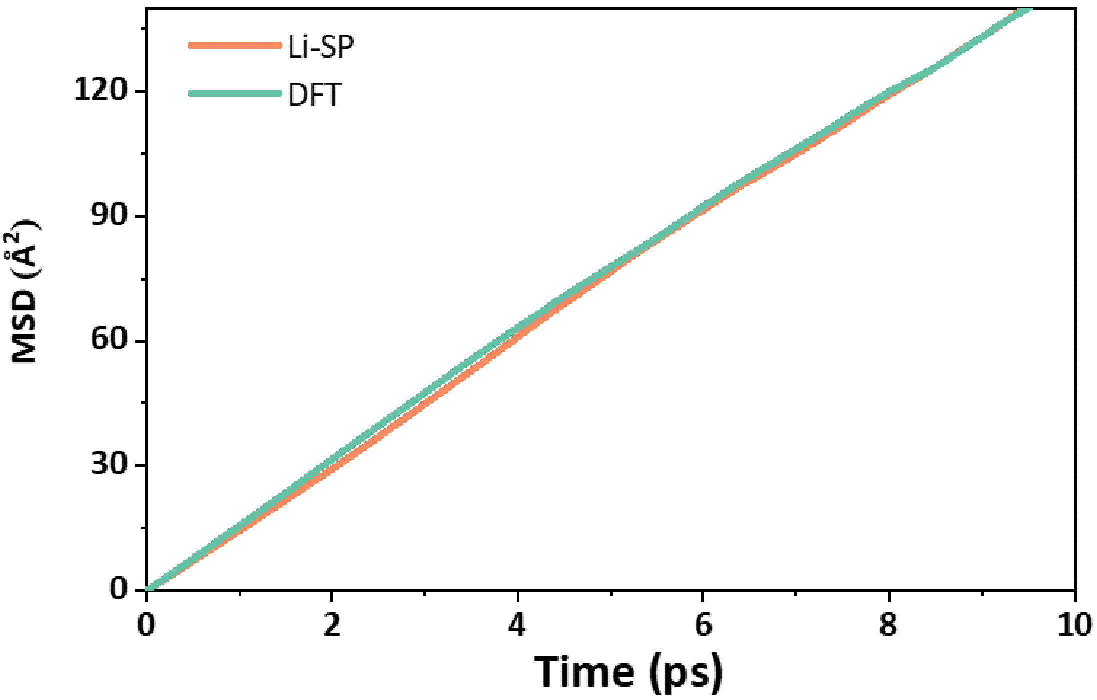

Figure S7. MSD calculated by Li-SP and DFT at 1,000 K.

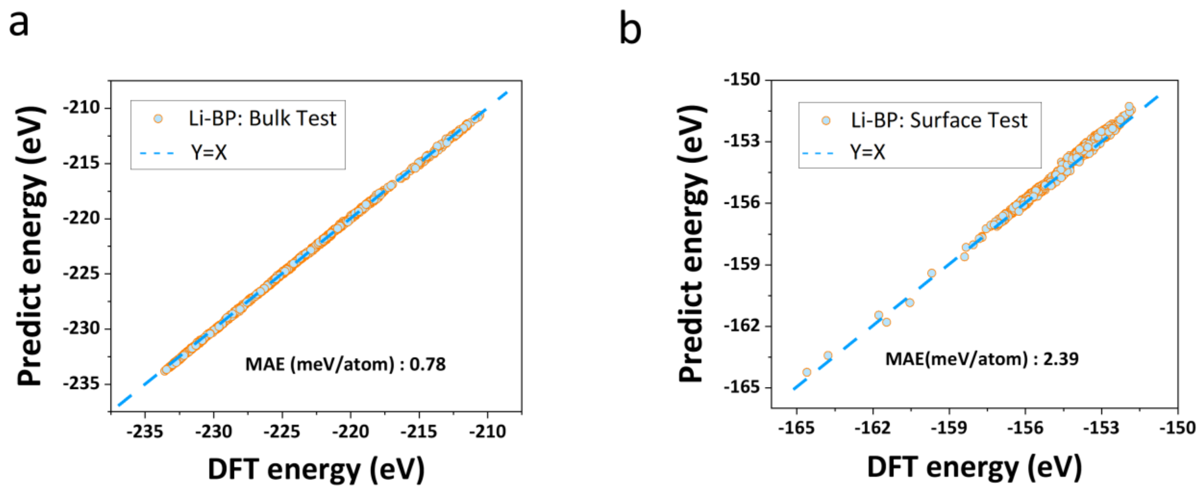

Figure S8. The energy test results predicted by lithium bulk potential (Li-BP) and DFT. **(a)** the results of bulk test dataset; (**b**) the results of the surface test dataset.

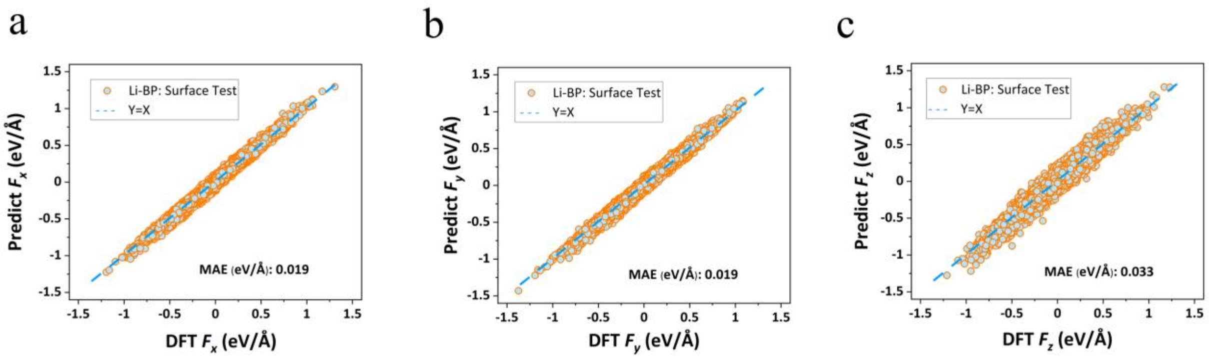

Figure S9. The force test of surface test dataset predicted by Li-DP and DFT calculation in three directions. (a) x, (b) y, and (c) z.

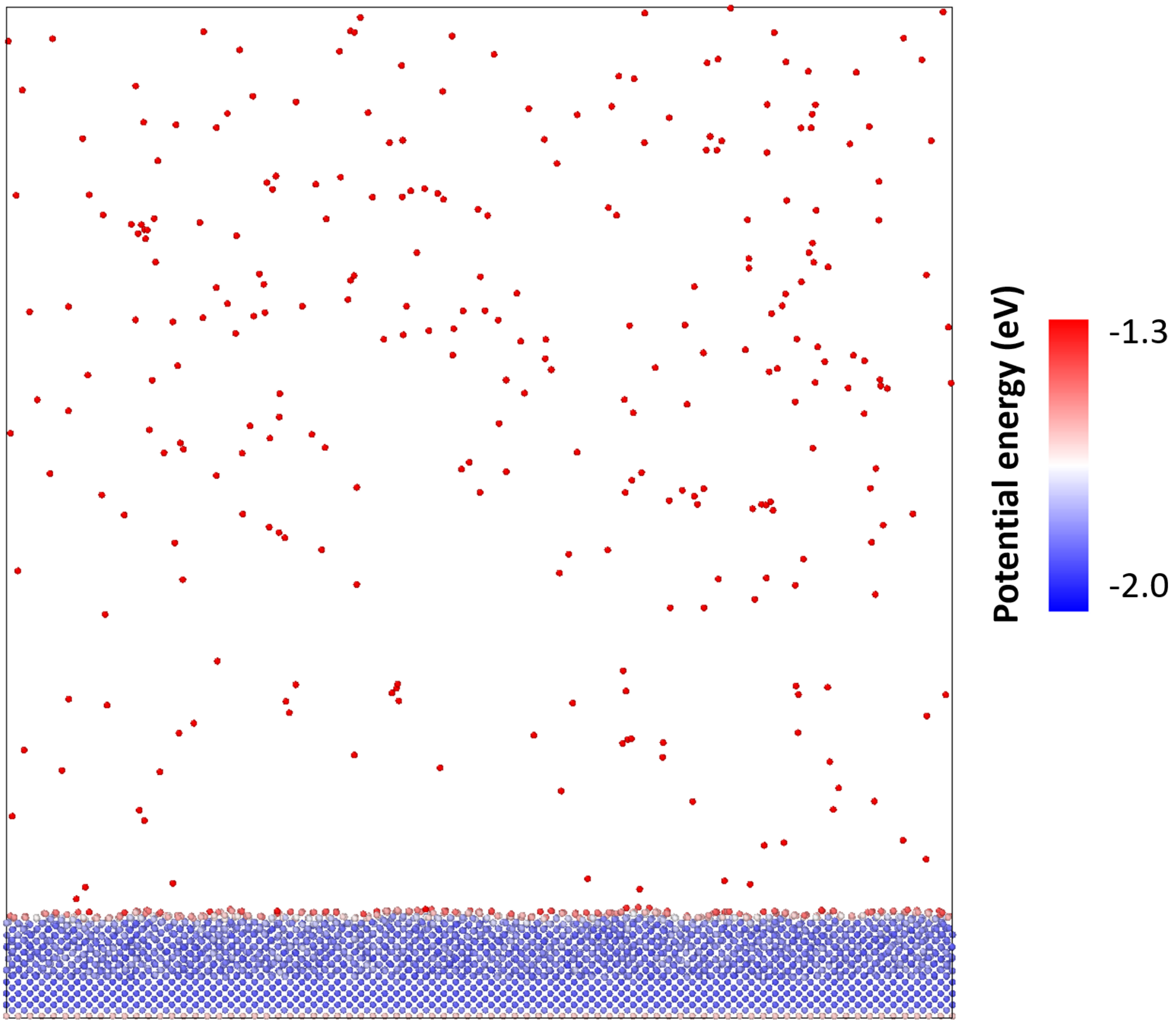

Figure S10. Schematic diagram of homogeneous deposition.

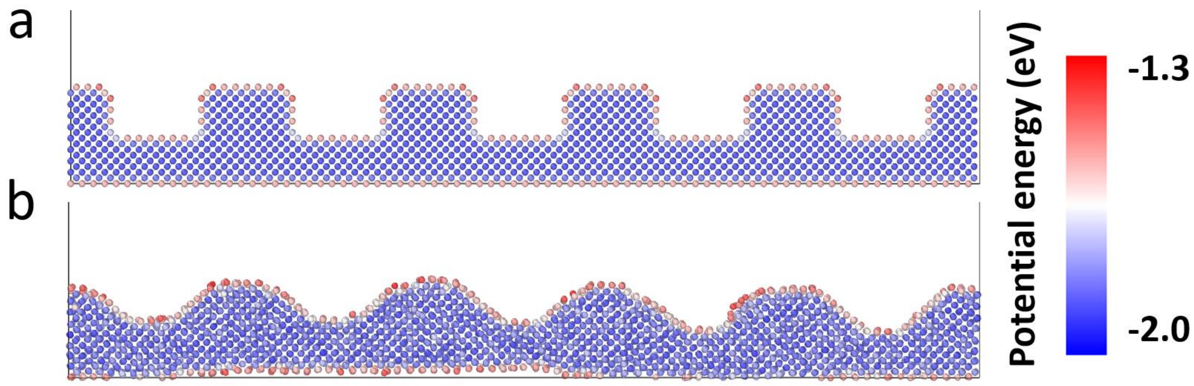

Figure S11. The evolution of the rectangular surface at 300 K.

(**a**) the initial configuration, (**b**) the configuration with a stable corrugated surface after relaxation.

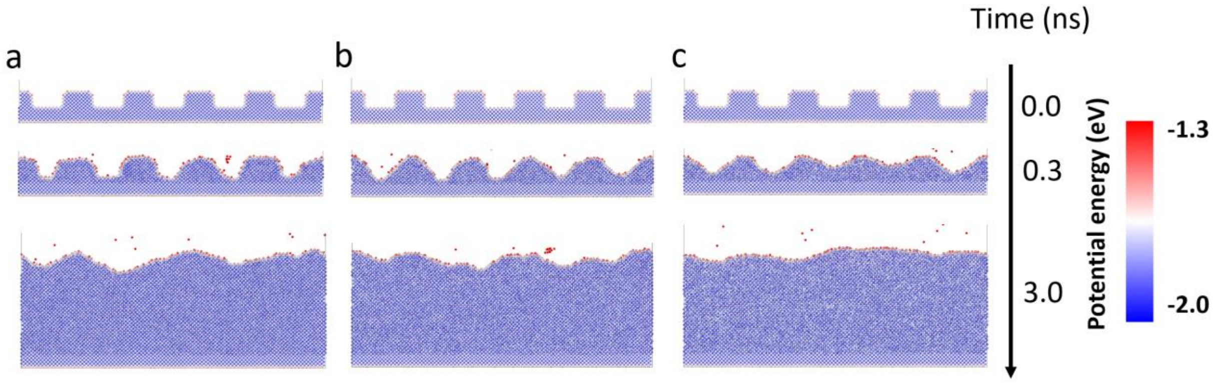

Figure S12. Snapshots of homogeneous deposition with a rectangular surface at different temperatures.
(**a**) 100 K, (**b**) 200 K, and (**c**) 300 K in NVT ensemble. The generation rate is 5 Li ps^{-1}.

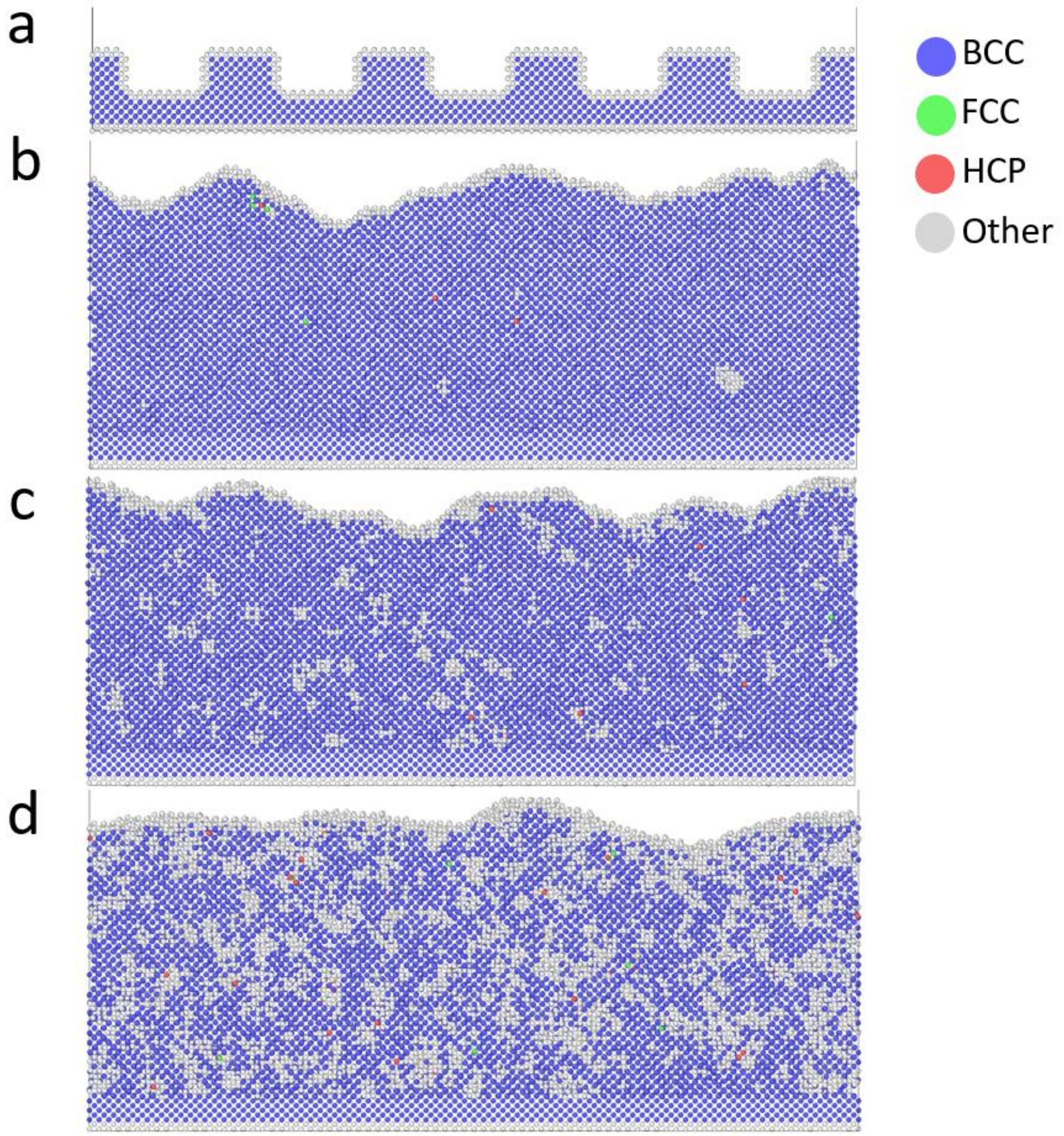

Figure S13. The Simulated results of homogeneous deposition at different temperatures by adaptive common neighbor analysis (a-CNA).

(**a**) the initial configuration, (**b**) the snapshot at 3 ns and 100 K, (**c**) the snapshot at 3 ns and 200 K, and (**d**) the snapshot at 3 ns and 300 K. The blue, green, and red balls present body-centered cubic (BCC), face-centered cubic (FCC), and hexagonal close-packed (HCP), respectively. The gray ball presents other local environments, including surface and amorphous atoms.

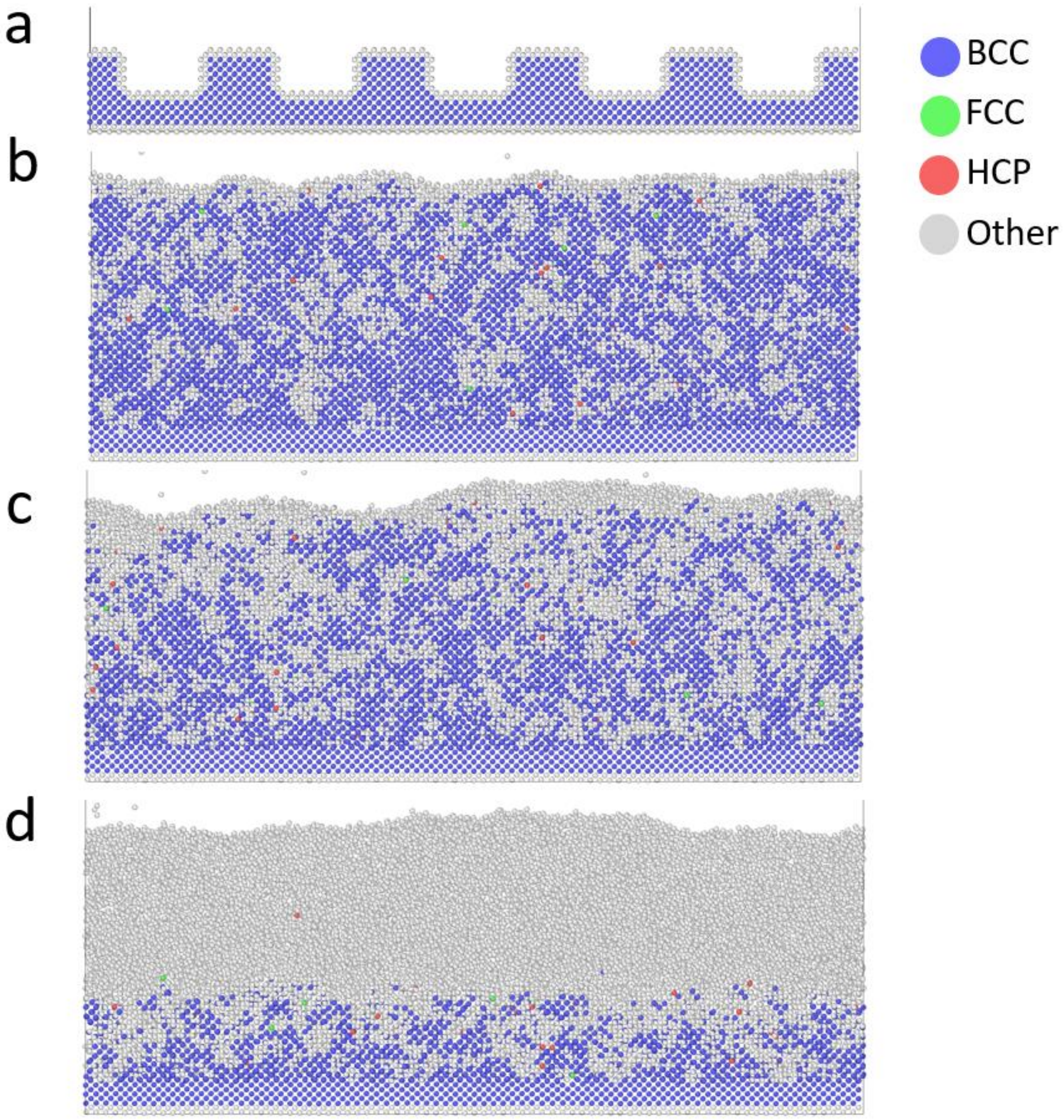

Figure S14. Simulated results of homogeneous deposition at different deposition rates. (**a**) 1 Li ps^{-1}, 15 ns (**b**) 5 Li ps^{-1}, 3 ns (**c**) 20 Li ps^{-1}, 0.75 ns. The number of deposition Li atoms is 15000. The surface of the initial structure is smooth, and the temperature is 300 K.

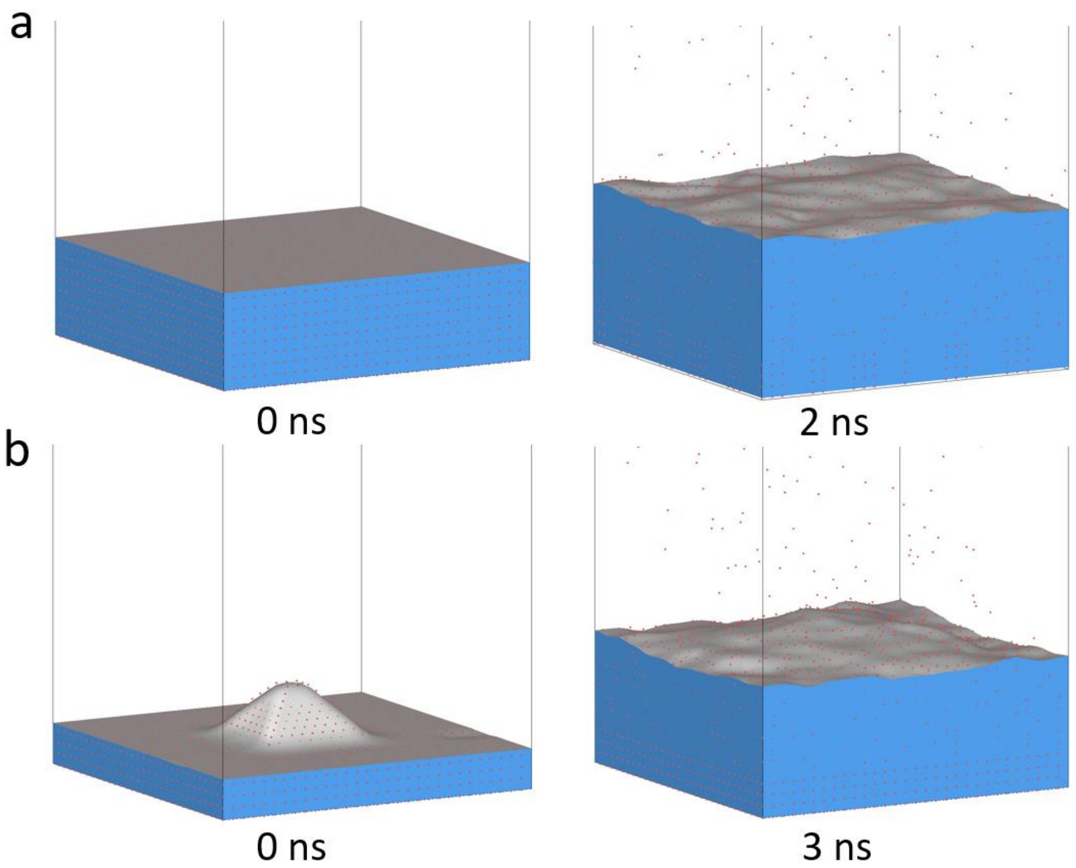

Figure S15. Simulated results of Li deposition under the configuration of a box of 10.5 nm (x) × 10.5 nm (y) × 30.0 nm (z) at 300 K, and the deposition rate was 5 Li ps^{-1}. (**a**) flat surface, (**b**) positive triangle surface.

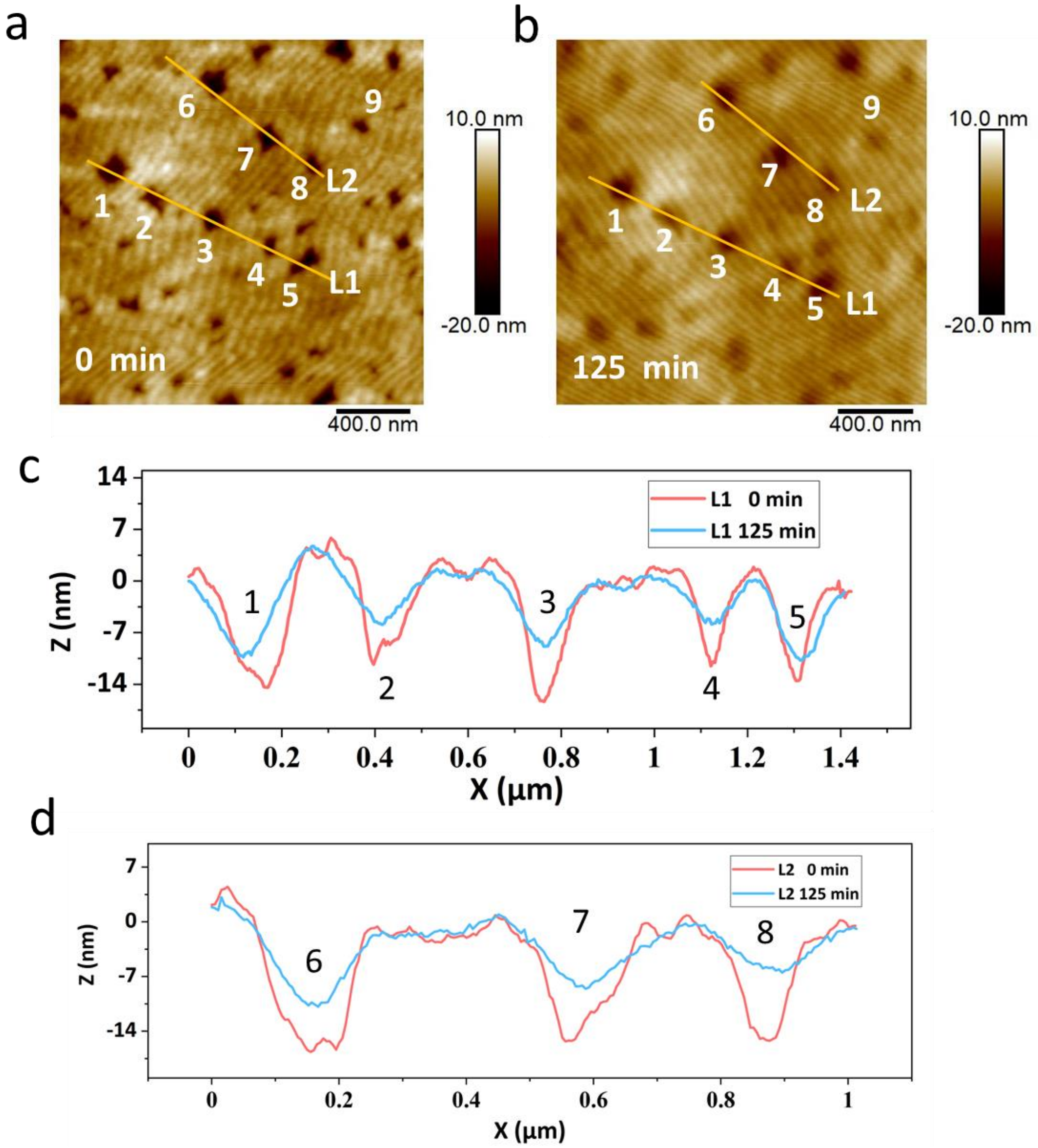

Figure S16. Atomic force microscope images (AFM) of Li foil with surface defects. (a) 0 min, (b) 125 min, the depth of defects line L1 (c) and L2 (d) at different times. The defect ID numbers in (c) and (d) correspond to the ID numbers in (a) and (b). The colors in the color bar represent the depth of the defects.

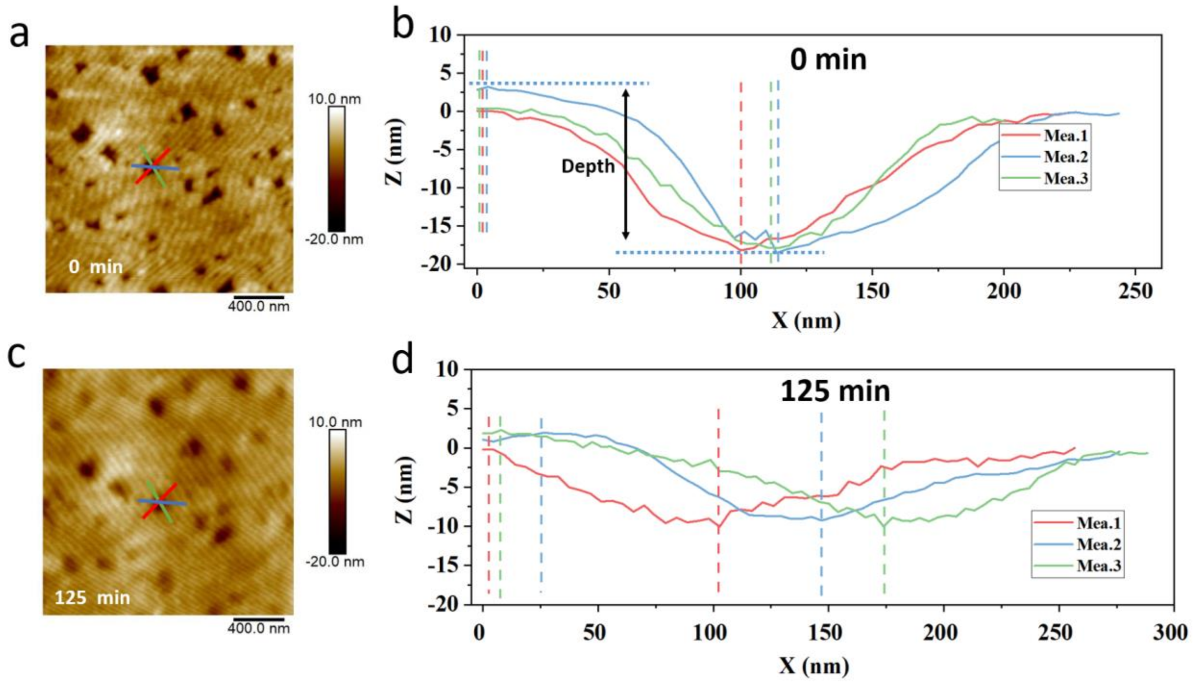

Figure S17. Illustration of defects depth measurement. (a) AFM image at 0 min,(b) three lines for defects depth measurements at 0 min, (c) AFM image at 125 min, and (d) three lines for defects depth measurements at 125 min.

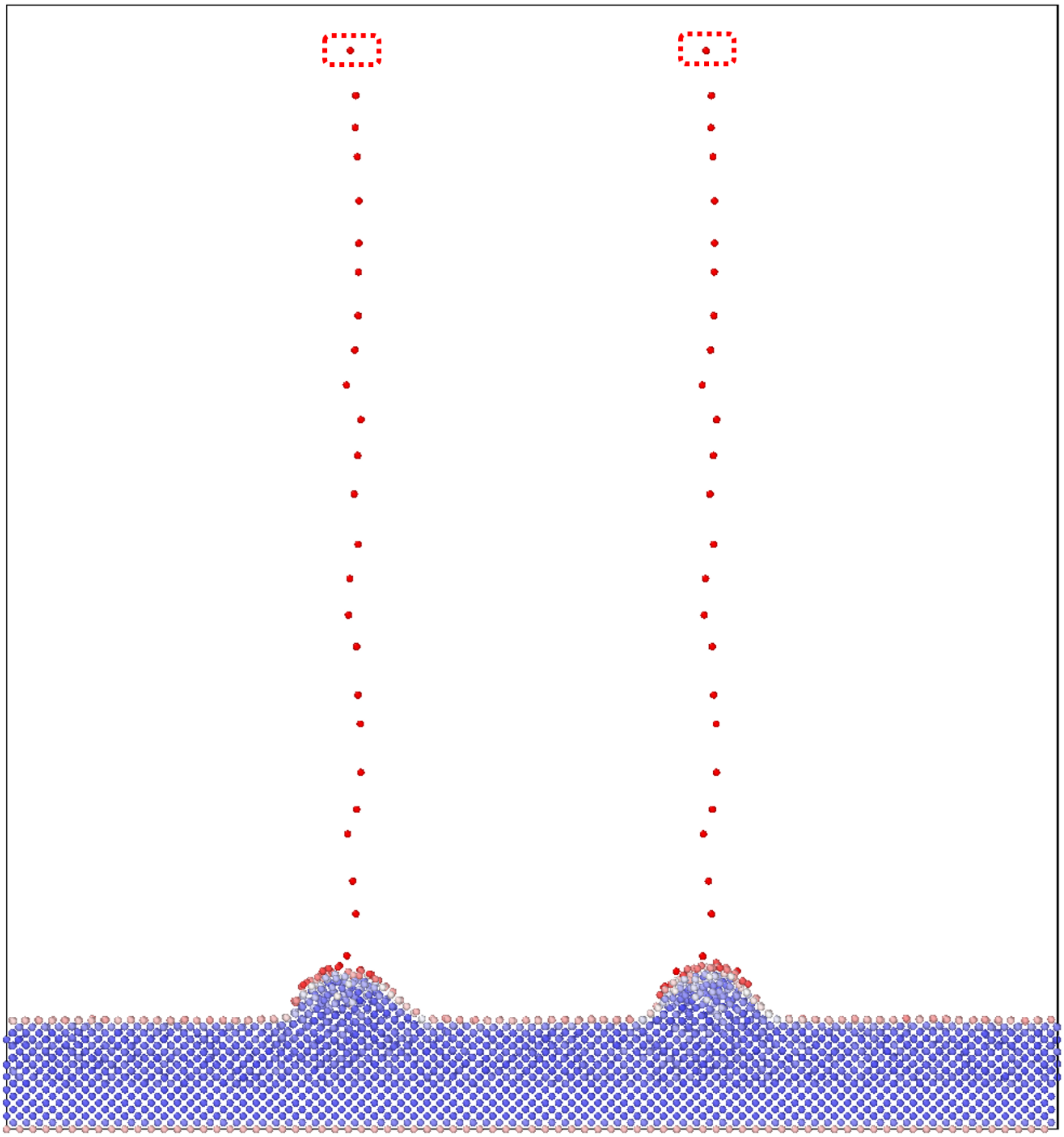

Figure S18. Schematic diagram of inhomogeneous deposition. Li atoms are generated in the red (rectangle) areas.

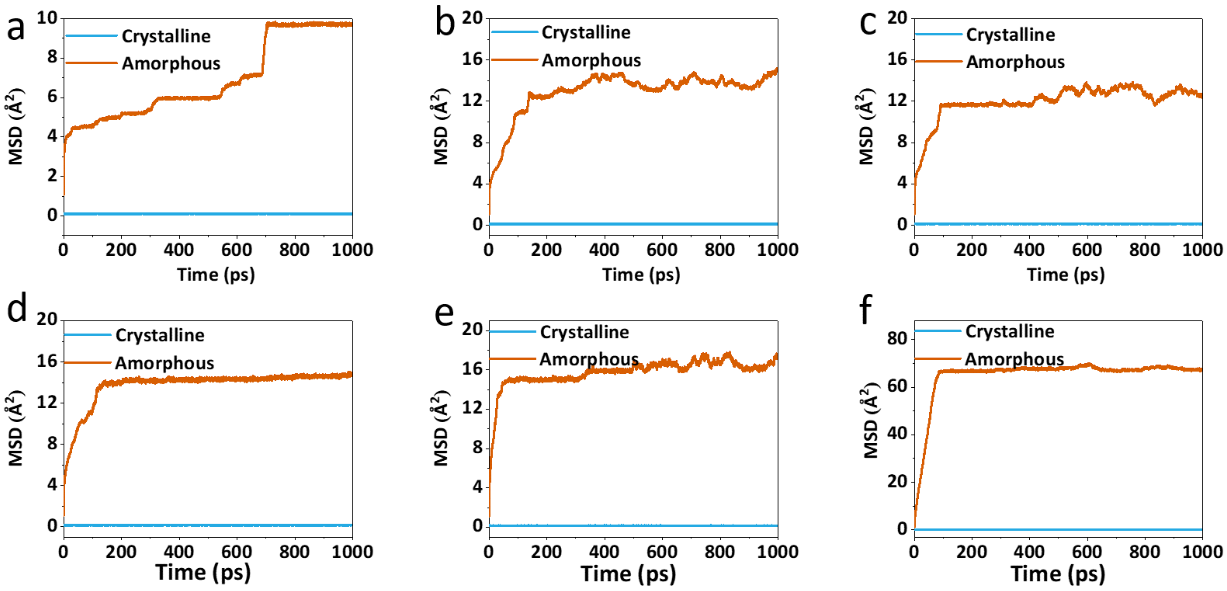

Figure S19. Mean square displacement comparison (MSD) of amorphous state and crystalline state (initial structure) at different temperatures.

(**a**) 50 K; (**b**) 100 K; (**c**) 150 K; (**d**) 200 K; (**e**) 250 K; (**f**) 300 K. The initial crystalline configuration contains 2,000 Li atoms with a 10×10×10 of pristine BCC structure. The initial amorphous configuration was obtained by MD of the initial crystalline configuration at 1,000 K.

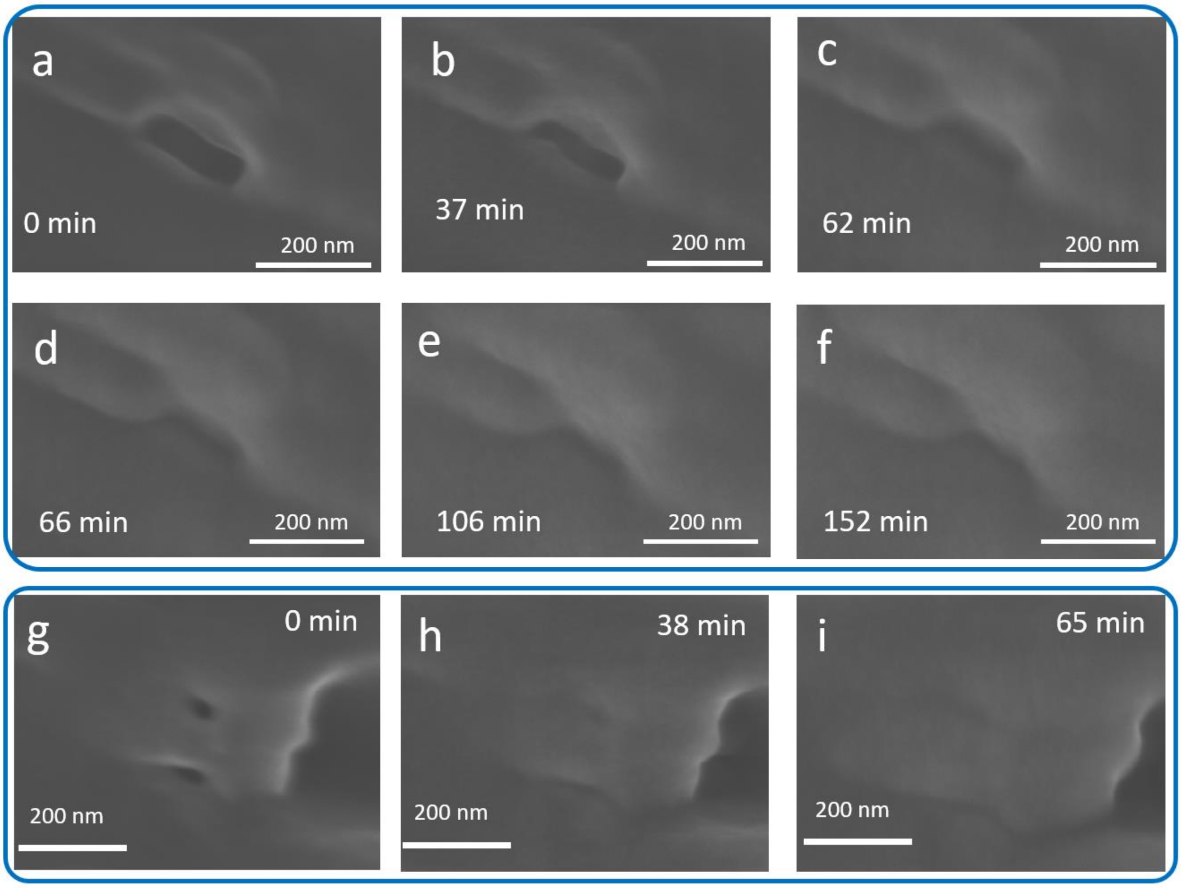

Figure S20. Li bulk self-healing process under the scanning electron microscope.

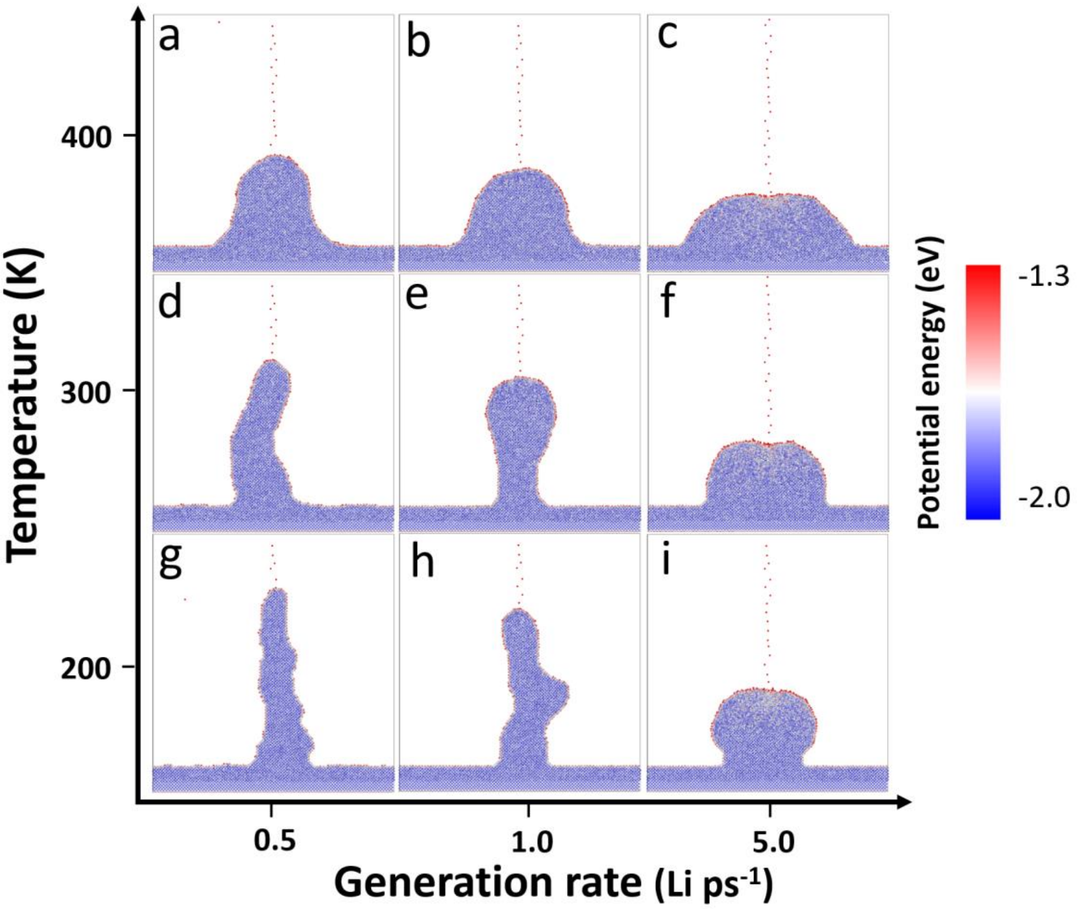

Figure S21. The snapshots of the inhomogeneous deposition with ~6000 deposited Li atoms in different temperatures and generation rate.

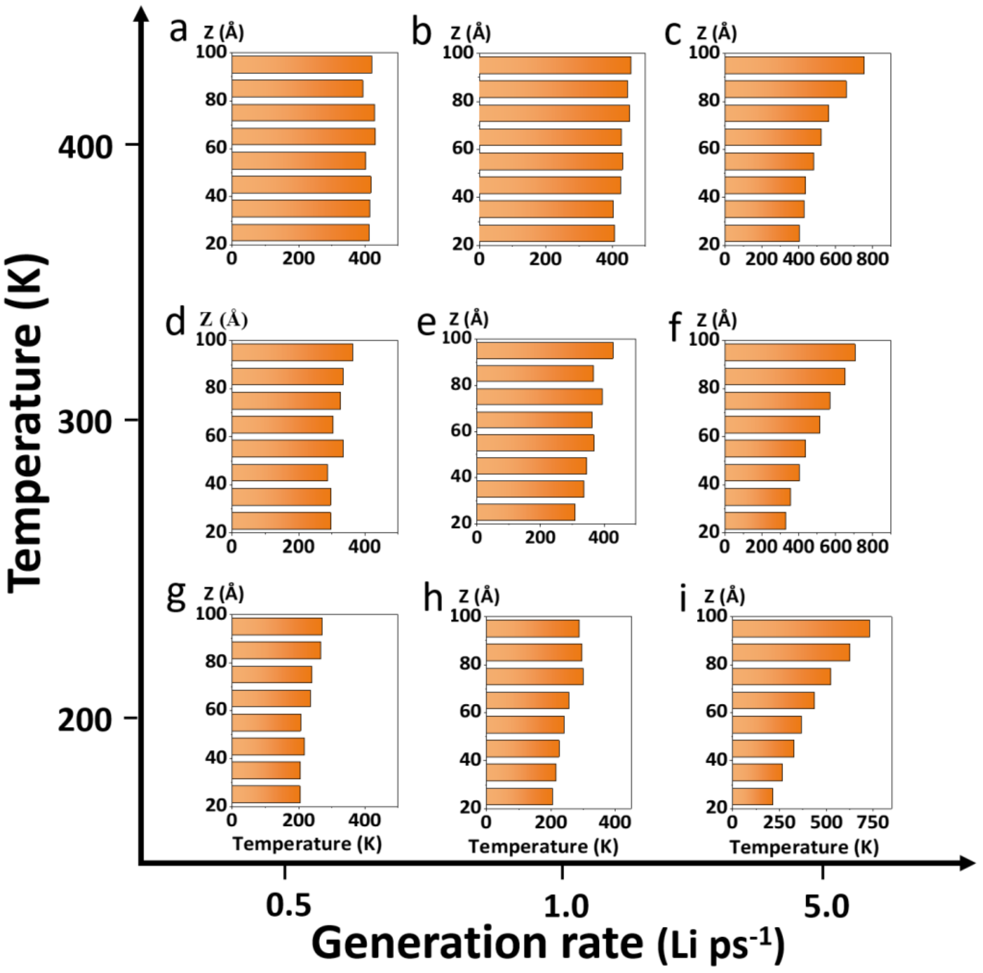

Figure S22. The temperature at different dendrite altitudes corresponding to Figure 3. The local temperatures of the dendrite were calculated by dividing the dendrite into several sub-regions according to the size of the Z-axis. The divided interval of the Z-axis is 1 nm.

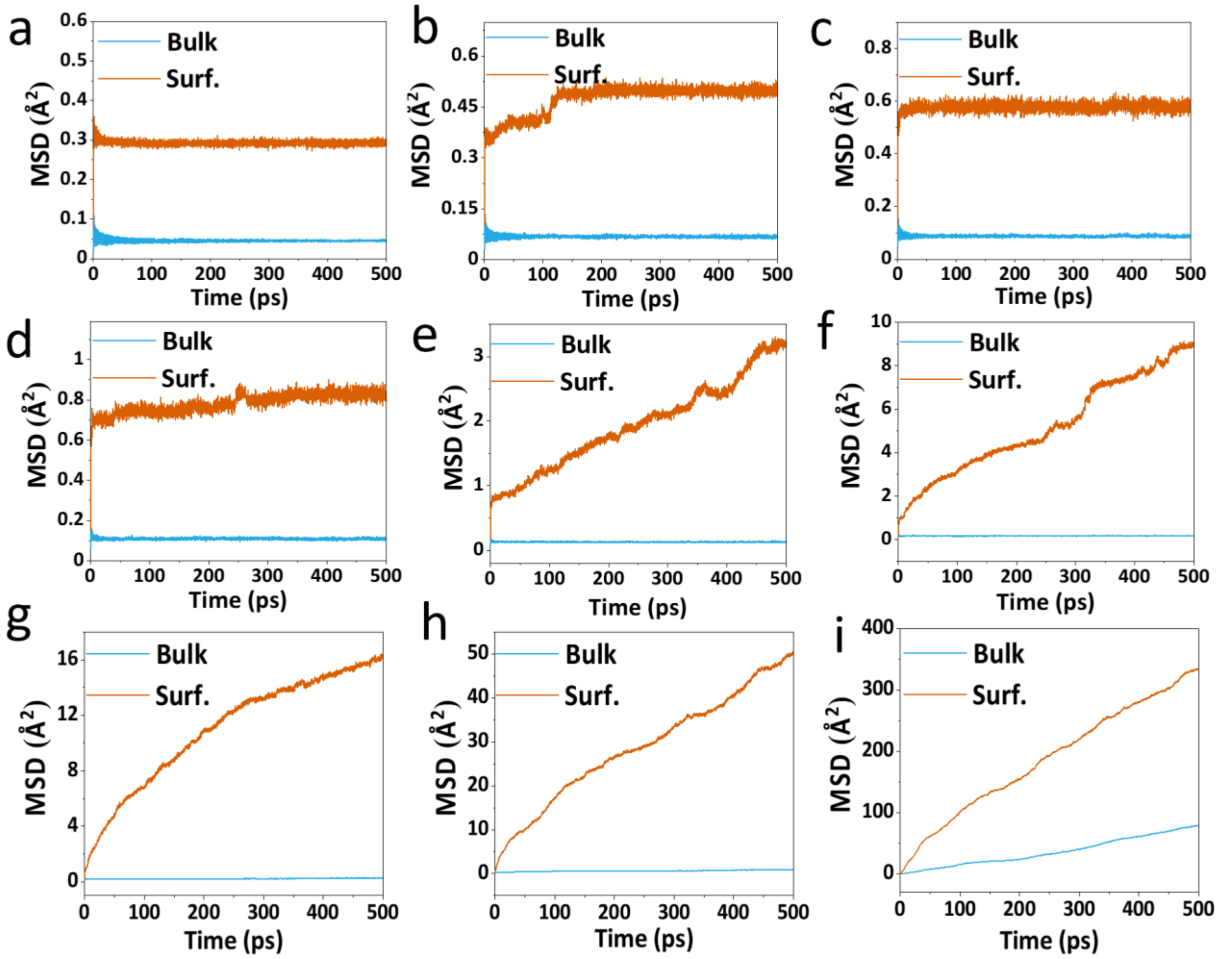

Figure S23. MSD for bulk and surface atoms at different temperatures.
(**a**) 50 K, (**b**) 100 K, (**c**) 150 K, (**d**) 200 K, (**e**) 250 K, (**f**) 300 K, (**f**) 350 K, (**h**) 400 K, and (**i**) 450 K.

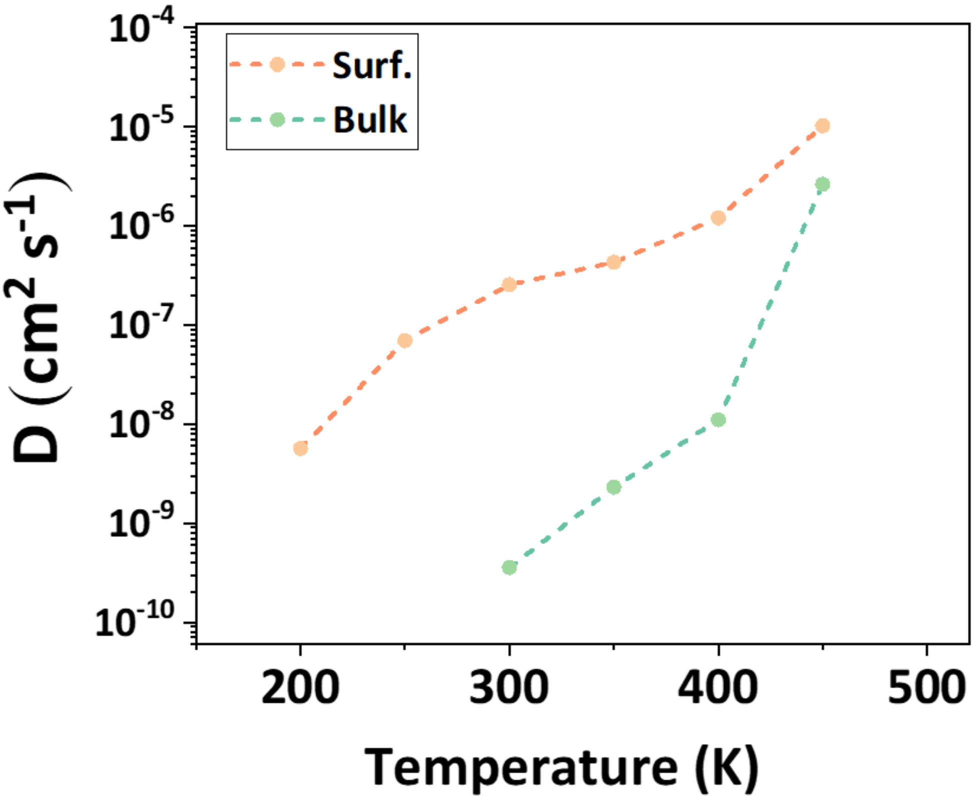

Figure S24. The diffusion coefficient of surface and bulk Li atoms with different temperatures.

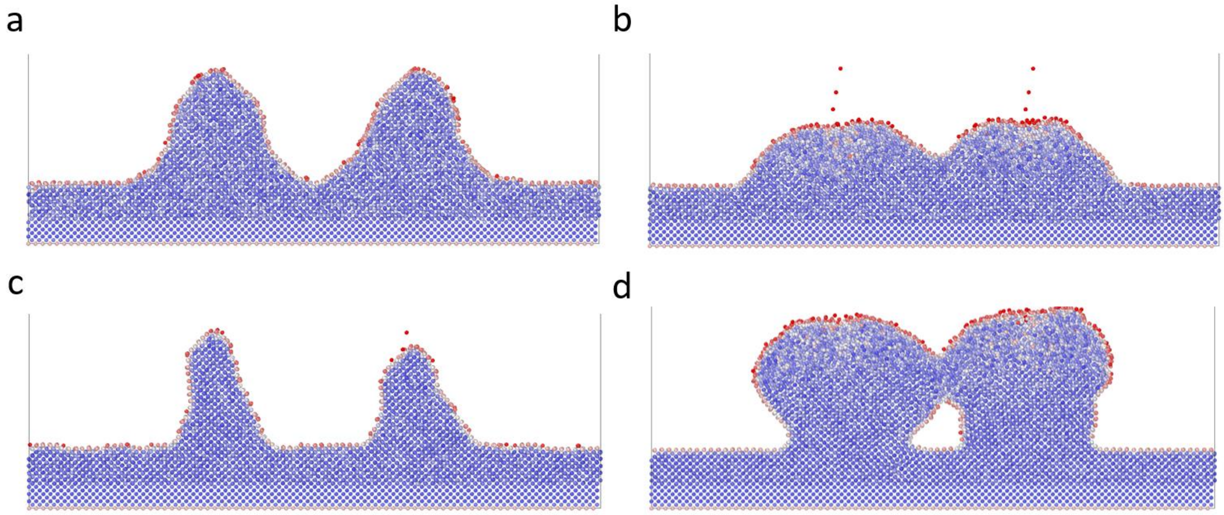

Figure S25. The schematic diagram of bulk self-healing in different temperatures and Gr.

(**a**) 350 K at 1 Li ps^{-1}; (**b**) 300 K at 5 Li ps^{-1}; (**c**) 200 K at 1 Li ps^{-1}; and (**d**) 100 K at 5 Li ps^{-1}.

Supplementary Tables

Table S1. Physical properties of Li predicted by various models.

The physical properties include elastic constants (C_{ij}), bulk modulus (B_v), shear modulus (G_v), surface energy with three low Miller indexes, and the melting point (T_m). The test models are DFT, EAM, MEAM, Li-SP, Li-BP, and experiments. The Li-SP did not show obvious advantages when compared with EAM and MEAN in the calculation of some elastic constants for the reason that our training datasets have no information about elastic constants and modulus, while both EAM and MEAN take elastic constants and modulus as the targets to fit the potential parameters [11, 12].

		DFT	EAM	MEAM	Li-SP	Li-BP	Exp.
**Elastic (GPa)**	C_{11}	15 (a)	14.74	14.96	16.36	19.45	14.8 (b)
	C_{12}	13 (a)	12.49	12.45	11.72	14.46	12.5 (b)
	C_{44}	11 (a)	10.73	10.33	10.87	12.02	10.8 (b)
**Modulus (Gpa)**	B_V	14 (a)	12.57	13.29	13.27	16.12	/
	G_V	7 (a)	6.89	6.70	7.45	8.21	/
**Surface Energy (J/m^2)**	111	0.54 (c)	0.460	0.473	0.545	0.575	/
	110	0.50 (c)	0.354	0.404	0.481	0.487	/
	100	0.46 (c)	0.361	0.397	0.463	0.503	/
**T_m (K)**		/	660 (d)	450 $\pm$ 10 (e)	451.6	445	454 (f)

a. ***ref.*** [21]
b. ***ref.*** [12, 14]
c. ***ref.*** [22]
d. ***ref.*** [11]
e. ***ref.*** [12]
f. ***ref.*** [13]

Table S2. The depth measurement for Li surface defects based on AFM images.
The measurements were carried out at 0 min and 125 min, respectively, after the Li surface defects were made. At each time, three independent measurements (Figure S17) were carried out to produce an accurate result and the average of the three measurements was calculated (Average column). The Defects ID is corresponding to Figure S16.

Defects ID	Depth at 0 min (nm)				Depth at 125 min (nm)			
	Mea.1	Mea.2	Mea.3	Average	Mea.1	Mea.2	Mea.3	Average
1	13.27	14.20	13.22	13.56	11.68	12.40	10.07	11.38
2	14.82	15.80	16.33	15.65	10.15	10.79	7.97	9.64
3	18.04	21.33	18.20	19.19	9.82	10.07	12.33	10.74
4	13.13	15.78	15.33	14.75	7.40	6.39	6.29	6.70
5	17.61	14.26	15.37	15.75	9.63	8.79	7.78	8.73
6	20.89	17.47	16.53	18.30	11.67	11.03	13.79	12.16
7	20.53	18.50	18.48	19.17	9.32	10.28	9.25	9.61
8	13.46	12.55	14.19	13.40	4.91	5.32	4.85	5.03

Supplementary References

[1] G. Kresse, J. Furthmüller, Phys. Rev. B 1996, 54, 11169; G. Kresse, J. Furthmüller, Comput. Mater. Sci. 1996, 6, 15.
[2] J. P. Perdew, K. Burke, M. Ernzerhof, Phys. Rev. Lett. 1996, 77, 3865.
[3] P. E. Blöchl, Phys. Rev. B 1994, 50, 17953.
[4] L. Zhang, J. Han, H. Wang, R. Car, W. E, Phys. Rev. Lett. 2018, 120, 143001.
[5] H. Wang, L. Zhang, J. Han, W. E, Comput. Phys. Commun. 2018, 228, 178.
[6] L. Zhang, D.-Y. Lin, H. Wang, R. Car, W. E, Phys. Rev. Mater. 2019, 3, 023804; Y. Zhang, H. Wang, W. Chen, J. Zeng, L. Zhang, H. Wang, W. E, Comput. Phys. Commun. 2020, 253, 107206.
[7] L. F. Zhang, J. Q. Han, H. Wang, W. A. Saidi, R. Car, W. N. E, in *Advances in Neural Information Processing Systems 31*, Vol. 31 (Eds: S. Bengio, H. Wallach, H. Larochelle, K. Grauman, N. CesaBianchi, R. Garnett), Neural Information Processing Systems (Nips), La Jolla 2018.
[8] K. M. He, X. Y. Zhang, S. Q. Ren, J. Sun, Proceedings of the IEEE Conference on Computer Vision and Pattern Recognition (CVPR) 2016, 770.
[9] D. P. Kingma, J. Ba, arXiv preprint arXiv:1412.6980 2014.
[10] J. Wu, Y. Zhang, L. Zhang, S. Liu, Phys. Rev. B 2021, 103, 024108.
[11] A. Nichol, G. J. Ackland, Phys. Rev. B 2016, 93, 184101.
[12] Z. Cui, F. Gao, Z. Cui, J. Qu, Modell. Simul. Mater. Sci. Eng. 2011, 20, 015014.
[13] E. A. Brandes, G. Brook, Eds., *Smithells Metals Reference Book*, Butterworth-Heinemann, Oxford, ed. 7, 1992.
[14] W.-S. Ko, J. B. Jeon, Comput. Mater. Sci. 2017, 129, 202.
[15] S. Plimpton, J. Comput. Phys. 1995, 117, 1.
[16] A. Stukowski, Modell. Simul. Mater. Sci. Eng. 2010, 18, 015012.
[17] A. Stukowski, Modell. Simul. Mater. Sci. Eng. 2012, 20, 045021.
[18] M. Yang, Y. Liu, A. M. Nolan, Y. Mo, Adv. Mater. 2021, 33, 2008081.
[19] X. Wang, G. Pawar, Y. Li, X. Ren, M. Zhang, B. Lu, A. Banerjee, P. Liu, E. J. Dufek, J.-G. Zhang, J. Xiao, J. Liu, Y. S. Meng, B. Liaw, Nat. Mater. 2020, 19, 1339.
[20] L. Li, S. Basu, Y. Wang, Z. Chen, P. Hundekar, B. Wang, J. Shi, Y. Shi, S. Narayanan, N. Koratkar, Science 2018, 359, 1513.
[21] M. de Jong, W. Chen, T. Angsten, A. Jain, R. Notestine, A. Gamst, M. Sluiter, C. K. Ande, S. van der Zwaag, J. J. Plata, C. Toher, S. Curtarolo, G. Ceder, K. A. Persson, M. Asta, Sci. Data 2015, 2.
[22] R. Tran, Z. H. Xu, B. Radhakrishnan, D. Winston, W. H. Sun, K. A. Persson, S. P. Ong, Sci. Data 2016, 3.
